# Synthesis and Characterization of Zn–Organic Frameworks Containing Chitosan as a Low-Cost Inhibitor for Sulfuric-Acid-Induced Steel Corrosion: Practical and Computational Exploration

**DOI:** 10.3390/polym14020228

**Published:** 2022-01-06

**Authors:** Mohamed Gouda, Mai M. Khalaf, Kamal Shalabi, Mohammed A. Al-Omair, Hany M. Abd El-Lateef

**Affiliations:** 1 Department of Chemistry, College of Science, King Faisal University, P.O. Box 400, Al-Hofuf 31982, Saudi Arabia; mmkali@kfu.edu.sa (M.M.K.); alomair@kfu.edu.sa (M.A.A.-O.); 2 Chemistry Department, Faculty of Science, Sohag University, Sohag 82524, Egypt; 3 Chemistry Department, Faculty of Science, Mansoura University, Mansoura 11432, Egypt; dr-kamal@mans.edu.eg

**Keywords:** metal organic framework (MOF), chitosan, duplex steel, solvothermal method, corrosion protection, theoretical study

## Abstract

In this work, a Zn–benzenetricarboxylic acid (Zn@H_3_BTC) organic framework coated with a dispersed layer of chitosan (CH/Zn@H_3_BTC) was synthesized using a solvothermal approach. The synthesized CH/Zn@H_3_BTC was characterized by Fourier transform infrared spectroscopy (FTIR), field emission scanning electron microscope (FESEM), thermal gravimetric analysis (TGA), and Brunauer, Emmett, and Teller (BET) surface area. The microscopic observation and the analysis of the BET surface area of CH/Zn@H_3_BTC nanocomposites indicated that chitosan plays an important role in controlling the surface morphology and surface properties of the Zn@H_3_BTC. The obtained findings showed that the surface area and particle size diameter were in the range of 80 m^2^ g^−1^ and 800 nm, respectively. The corrosion protection characteristics of the CH/Zn@H_3_BTC composite in comparison to pristine chitosan on duplex steel in 2.0 M H_2_SO_4_ medium determined by electrochemical (*E* vs. time, PDP, and EIS) approaches exhibited that the entire charge transfer resistance of the chitosan- and CH/Zn@H_3_BTC-composite-protected films on the duplex steel substrate was comparatively large, at 252.4 and 364.8 Ω cm^2^ with protection capacities of 94.1% and 97.8%, respectively, in comparison to the unprotected metal surface (*R*_p_ = 20.6 Ω cm^2^), indicating the films efficiently protected the metal from corrosion. After dipping the uninhabited and protected systems, the surface topographies of the duplex steel were inspected by FESEM. We found the adsorption of the CH/Zn@H_3_BTC composite on the metal interface obeys the model of the Langmuir isotherm. The CH/Zn@H_3_BTC composite revealed outstanding adsorption on the metal interface as established by MD simulations and DFT calculations. Consequently, we found that the designed CH/Zn@H_3_BTC composite shows potential as an applicant inhibitor for steel protection.

## 1. Introduction

The steel alloys utilized in oil and petroleum manufacturing can vary from API N80 mild steel, J55, and L80, to corrosion-resistant alloys with high chromium content, such as austenitic-ferritic steel, amended martensitic 13.0% Cr steel (Super-13), and duplex 20–22% Cr steel [1,2,3]. Corrosion is a serious problem affecting steel alloys in harsh solutions, particularly in acidic media, which hinders the lifetime and the related abilities of these alloys, and can even result in severe accidents. Additionally, the rust released from steel into the environment imposes a heavy ecological burden. Thus, researchers have focused on improving the deterioration of C steel in various harsh solutions.

Corrosion inhibitors are one of the experimental approaches used for protection against corrosion, specifically in acidic solutions [4,5,6,7,8]. The various inhibitors that have been applied and test industrially as corrosion additives [9,10,11,12] are those are low- or non-toxic are currently better than the materials used in the recent past such as glucose, gellan gum, carboxymethyl, hydroxypropyl cellulose [9,11], xanthan gum [10], and chitosan-g-PEG [12]. Recently, the research in the area of eco-friendly or green corrosion inhibitors has been directed toward fabricating inexpensive, efficient composites that have no or minimal environmental impact [13,14].

Organic bridging ligands and metal nodes are utilized to create metal–organic frameworks (MOFs) [15]. MOFs are gaining popularity among researchers because of their ease of functionalization, variable pore diameters, and large surface area [16,17]. MOFs are potential candidates for a variety of applications, including gas separation and storage [18], fuel cells [19], solar cells [20], catalysis [21], sensors [22], electrical devices [23], drug delivery [24], and others, due to their beneficial features.

Chitosan is made by deacetylating chitin via alkaline hydrolysis [25]. Chitosan is a biodegradable, biocompatible, nontoxic, and structurally stable biopolymer [26]. Chitosan-coated MOFs preserve their crystalline structure and high porosity and do not producing toxicity issues compared to pure MOFs [27]. MOFs’ chemical and colloidal stabilities may also be improved via surface modification.

As a result of the accuracy of MOF construction, scientists have continue to develop applications of MOF compounds and have accomplished many outcomes, but comparatively fewer studies have applied them for corrosion inhibition [28,29]. The MOF structure has large pores; consequently, sequences of composite compounds are fabricated using various groups on graphene oxide to coordinate with the MOF materials with the purpose of obstructing the characteristics of graphene, providing the MOF with corrosion protection ability, thereby inhibiting the impact of corrosion on steel [30,31,32]. The anticorrosion properties of MOF-decorated graphene oxide nano-platforms were investigated; the findings showed that the graphene oxide/zeolitic imidazolate framework-8 loaded composite material has respectable protection capacity [33]. A benzotriazole/MOF/tetraethyl orthosilicate/graphene oxide nanocomposite was prepared and its anticorrosive action was inspected. The findings confirmed that the fabricated nanocomposite combined into the protective films presented strong anticorrosion characteristics [34].

To overcome the problems due to the large pores in MOF skeletons, in this study, the prepared MOFs material was coated with chitosan as a dispersed layer. We synthesized chitosan containing a Zn@1,3,5-benzene tricarboxylic acid framework via the solvothermal method and characterized it by FTIR, SEM, TGA, and BET surface area. Then, the corrosion protection capacities of the prepared CH/Zn@H_3_BTC were compared to those of pristine chitosan for duplex steel in sulfuric acid solution by electrochemical methods (EIS, PDP, and *E*_OCP_ vs. time) with concentrations ranging from 20 to 200 ppm. To examine the adsorption kinetics of CH and CH/Zn@H_3_BTC on the metal substrate, some models of the adsorption isotherms were examined. The changes in the morphology of the corroding metal substrate in the blank and inhibited systems were detected by FESEM. Monte Carlo simulations and DFT calculations were concluded to understand the corrosion protection process.

## 2. Experimental Method

### 2.1. Materials

Chitosan with a high molecular weight with 85% deacetylation was purchased from Sigma-Aldrich Co. (St. Louis, MO, USA). Zn acetate dihydrate (ACS reagent, ≥98%) and 1,3,5-benzen tricarboxylic acid (98%) were purchased from Sigma-Aldrich Co. (St. Louis, MO, USA). Ethanol and other chemical reagents were purchased from Merck Co. (Darmstadt, Germany).

Duplex steel (D steel) was utilized during the experimentations. The chemical configuration of D steel is (wt.%): 0.012 C, 0.56 Si, 0.81 Mn, 0.019 P, 0.001 S, 22.15%Cr, 0.163 N, and 3.12 MO, and the rest is Fe. Before the corrosion protection a test, the working electrode (D steel) was abraded with a SiC paper in a sequence from 600 to 1500 grit. The samples were washed methodically with bidistilled H_2_O and degreased with acetone.

A stock solution of analytical-grade sulfuric acid (98%) was used to prepare an aqueous solution of the corrosive medium (2.0 M H_2_SO_4_) by the dilution process.

### 2.2. Preparation of CH/Zn@H_3_BTC Composite Beads

**Scheme 1:**
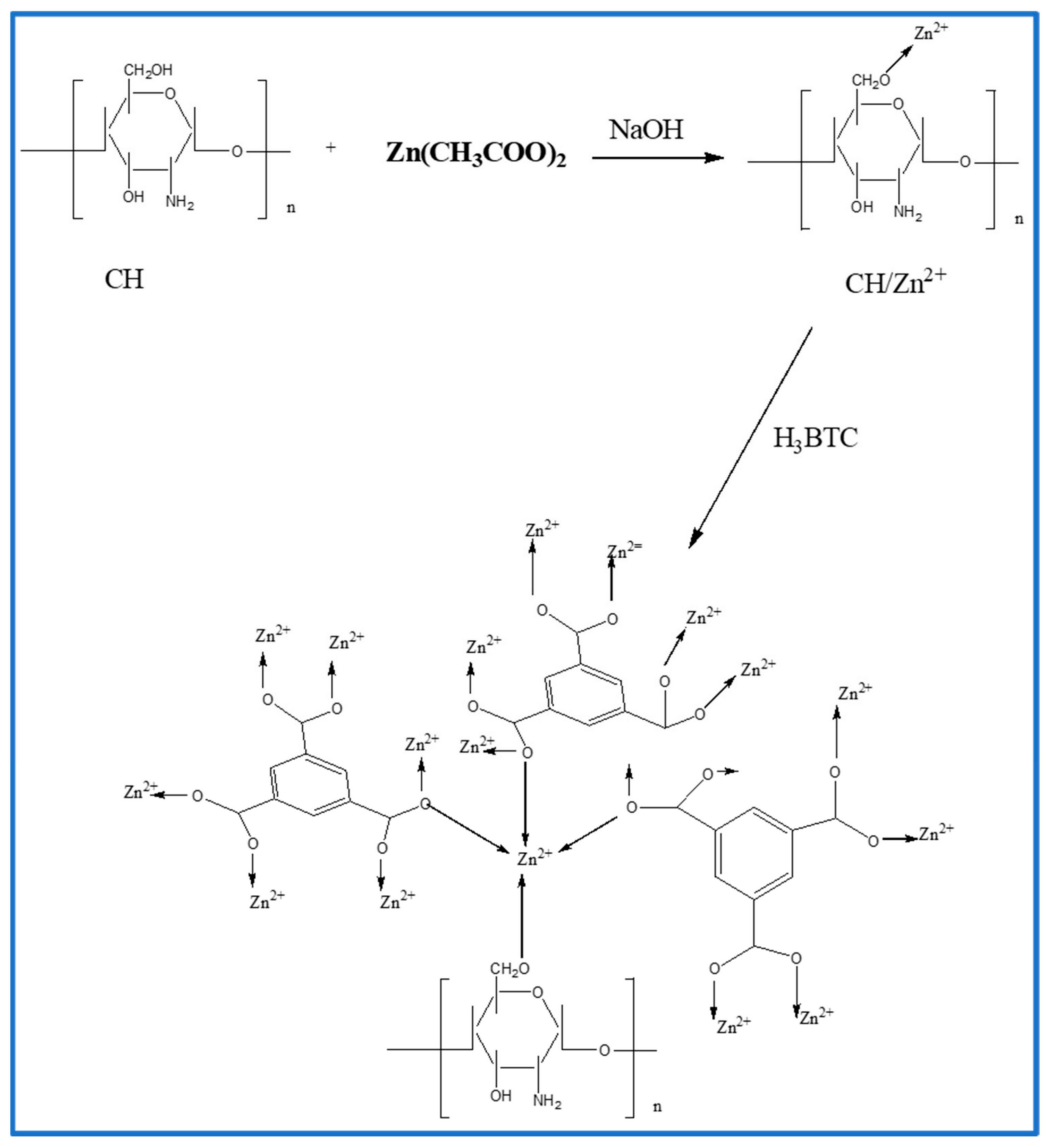
Proposed schematic synthesis of chitosan/Zn@1,3,5-benzen tricarbozylic acid (CH/Zn@H_3_BTC).

Scheme 1 depicts the CH/Zn@H_3_BTC composite bead preparation method. To make a homogenous solution, 1.0 g CH and 1.5 g zinc acetate were mixed in 100 mL acetic acid solution (1%, *v*/*v*) and stirred at 550 rpm for 4 h. The solution was then put into a 1.0 M NaOH solution. The CH/Zn^2+^ particles were removed after 15 min and rinsed three times with deionized water to remove the excess NaOH before being immersed in an aqueous solution containing 2.6 g (0.15 mol) 1,3,5-benzentricarboxylic acid (H_3_BTC). Zn^2+^ reacted with H_3_BTC at this point, forming Zn@H_3_BTC composite beads. The CH/Zn@H_3_BTC composite beads were then washed three times with deionized water, immersed in isopropanol, and freeze-dried for 12 h to produce CS/Zn@H_3_BTC composite beads.

### 2.3. Materials’ Characterization

A Fourier transform infrared (FTIR) spectrometer (Spectrometer Model FTIR-8400S, Shimadzu, Japan) was utilized in the range of 400.0 to 4000.0 cm^−1^ for the determination of the functional groups in the samples. Thermogravimetric analysis (TGA) was completed using a thermogravimetric device (Model-Q 500- Lukens Drive, TA Instruments, New Castle, DE, USA) to measure the thermal steadiness of CH/Zn@H_3_BTC. Field emission scanning electron microscopy (FESEM) (Model-JOEL-JXA-840-Tokyo 196-8558, Japan) was used to inspect the surface morphology of CH, Zn@H_3_BTC, and CH/Zn@H_3_BTC.

### 2.4. Specific Surface Area Studies

The surface area of CH/Zn@H_3_BTC were calculated using the Brunauer–Emmett–Teller (BET) method. The N_2_ adsorption–desorption was measured on a programmed gas sorption analyzer (Ji Nan RunZhi Technology, Shanghai, China). The prepared CH/Zn@H_3_BTC was outgassed for 4.0 to 6.0 h and N_2_ adsorption–desorption isotherms were evaluated at 77.35 K by the volumetric method, which represents extra adsorption isotherms [35].

### 2.5. Corrosion Protection Evaluation

The Gamry Galvanostat/Potentiostat/ZRA electrochemical workstation (Gamry Instruments, Warminster, PA, USA), according to the ASTM standard approach [36], was used to complete all electrochemical tests in a 3-electrode cell at 50 °C and under nonstirring conditions. A D steel substrate covered in an epoxy container (an efficient area of 1.0 cm^2^) was used as the operational electrode, Pt sheet was employed as the counter electrode, and silver/silver chloride (KCl_sat_.) was utilized as the reference electrode. Before the electrochemical tests, the working electrode (D steel) was immersed in the blank and inhibited corrosive medium for 45 min to steady the open circuit potential (*E*_ocp_). Potentiodynamic polarization (PDP) was performed with a potential range of −250 to + 250 mV vs. *E*_ocp_ at a scanning rate of 0.2 mV s^−1^. Electrochemical impedance spectroscopy (EIS) was completed in the frequency range of 0.1 Hz to 100 kHz with a sine amplitude of 10 mV at *E*_ocp_. Corrosion current density (*j*_cor_) was determined by the extrapolation of anodic and cathodic Tafel lines to corrosion potential (*E*_cor_) [6].

### 2.6. Surface Exploration

The surface topographies of the D steel substrates after 24 h of soaking in 2.0 M H_2_SO_4_ medium without and with 200 ppm of CH and CH/Zn@H_3_BTC at 323 K were scanned by an SEM device (JSM-6610 LV model) at an accelerating voltage of 20 kV. A surface investigation of the pristine polished D steel was also performed for comparison.

### 2.7. Theoretical Approaches

The correlation between the inhibition efficiencies and the molecular structure of the inhibitor was detected via theoretical calculations [37]. Computerized studies were performed by Accelrys Materials Studio 7.0 with the DMol^3^ module for DFT computations and with the adsorption locator module for MC simulations. Monomer structures of CH and CH/Zn@H_3_BTC were optimized in DFT calculations by applying the GGA/BLYP function with the DNP foundation set and COSMO solving controls [37]. For MC simulations, the suitable adsorption configurations for the monomer structures of CH and CH/Zn@H_3_BTC molecules on Fe (1 1 0) exterior were achieved via the adsorption locator module based on Monte Carlo investigations to assess the protection potency of the examined inhibitors [38]. The adsorption of the examined components, water particles, and exterior of Fe (1 1 0) was determined via a simulation box (32.27 × 32.27 × 50.18 Å^3^) through the appointed COMPASS force field [38].

## 3. Results and Discussions

### 3.1. Characterization Analysis

#### 3.1.1. FTIR

**Figure 1:**
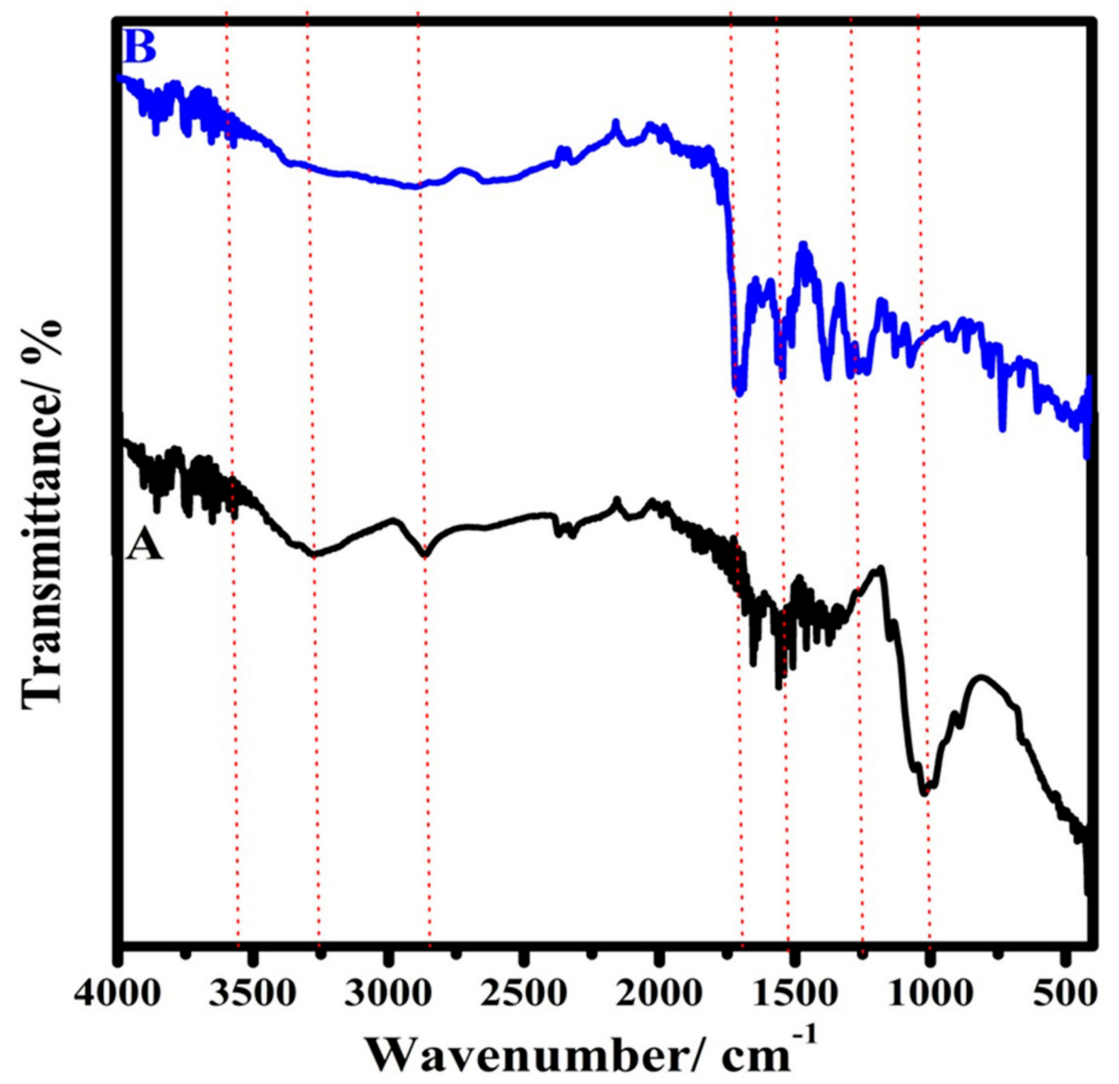
FTIR spectra of (**A**) CH and (**B**) CH/Zn@H_3_BTC.

Figure 1A,B shows the FTIR of blank CH and Zn@H_3_BTC/CH; the spectra show that the absorption bands of the carbonyl (C=O) and hydroxyl (–OH) groups. The peak observed at 1622 cm^−1^ matches the C=O stretching band and the peak at 1375 cm^−1^ was assigned as the stretching vibration the C–O bond, demonstrating the deprotonation of the carboxylate group (from H_3_BTC) and coordination with the Zn(II) metal ion to form ZnO_3_(BTC)_2_. We observed the characteristic peaks of the MOF at 1578 cm^−1^, corresponding to the C=C of the aromatic ring, and at 1521 cm^−1^, corresponding to the C=O of the strong carbonyl group. In addition, a characteristic peak at 1375 cm^−1^ was observed due to the presence of the carboxyl group. Due to the presence of carboxylate, no peak was observed at 1722 cm^−1^ due to the shift of the –OH group. This is mainly the reason that lengthy conjugate π-bonds are created from the carboxylate formed from carboxyl anion, thus creating the two-oxygen-atom equivalent. Furthermore, a strong characteristic peak was observed at 729 cm^−1^, corresponding to ZnO. According to Scheme 1, the zinc ion has four coordinated bonds, three of which are with H_3_BTC and the other with the chitosan molecule. Every H_3_BTC binds six zinc ions.

#### 3.1.2. Thermal Analysis

**Figure 2:**
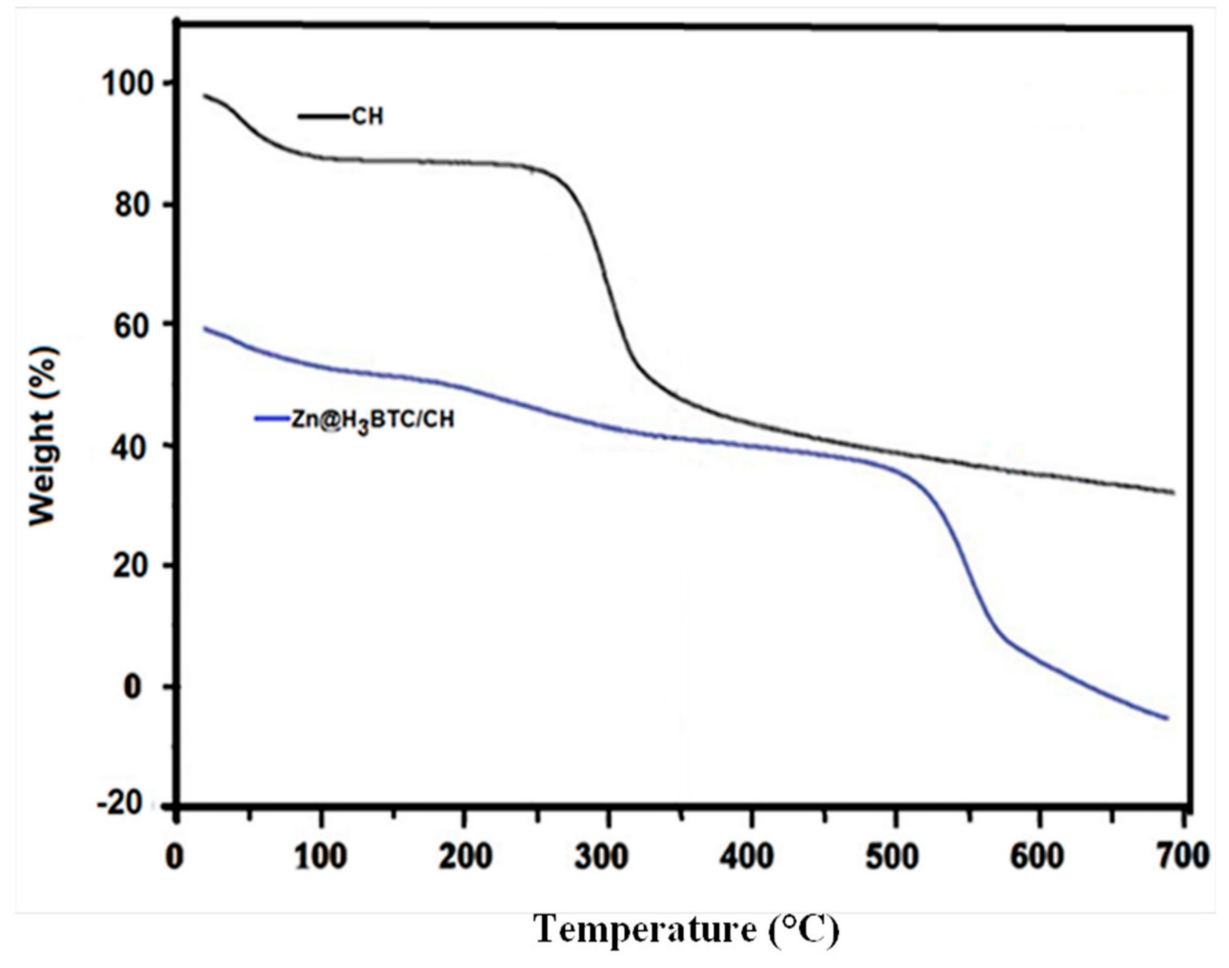
Thermogravimetric diagram of CH and CH/Zn@H_3_BTC.

Figure 2 displays the thermogravimetric measurement of CH and CH/Zn@H_3_BTC, illustrating the thermal stability and features of the thermal degradation of CH polymer and its composite. The overall weight loss of CH and CH/Zn@H_3_BTC, recorded with heating over the temperature range of RT–700 °C, were 40% and 60%, respectively. The results showed that the weight loss process and evolution of the CH and CH/Zn@H_3_BTC structures occurred through three steps stages of decomposition.

Based on the steps and the weight loss values for the CH sample and its composite, we assigned the first step below 120 °C to the loss of the adsorbed and included water and/or organic molecules; a ΔW of 10% was exhibited. This was followed by two steps from 200 to 380 °C, and a final step above 400 °C. The weight loss change for the CH sample for the second stage (ΔW = ~25%) and for the last stage (ΔW = ~5%) might have occurred due to the breakdown of chitosan molecules. The weight loss change for the CH/Zn@H_3_BTC sample was lower in the second stage (ΔW = ~7%) than in the last stage (ΔW = ~43%). The weight loss values assigned for the last stage of the CH sample (ΔW = ~5%) and the CH/Zn@H_3_BTC sample (ΔW = ~43%) confirm the insertion of Zn into the organic framework. Furthermore, the TGA curve of the CH/Zn@H_3_BTC had a larger weight loss compared to that of CH, thus confirming the improved interfacial binding of Zn into the chitosan matrix.

#### 3.1.3. FESEM

**Figure 3:**
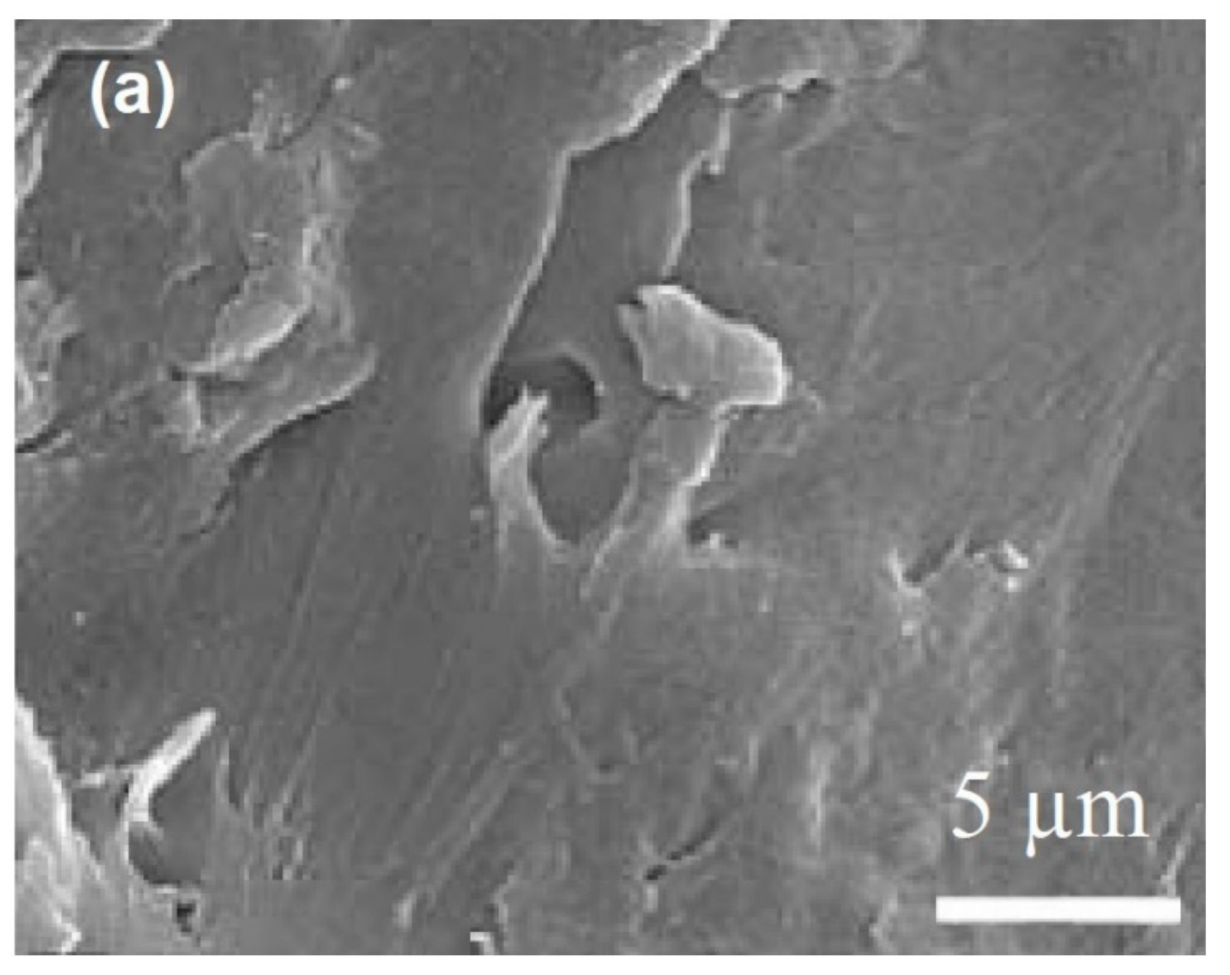

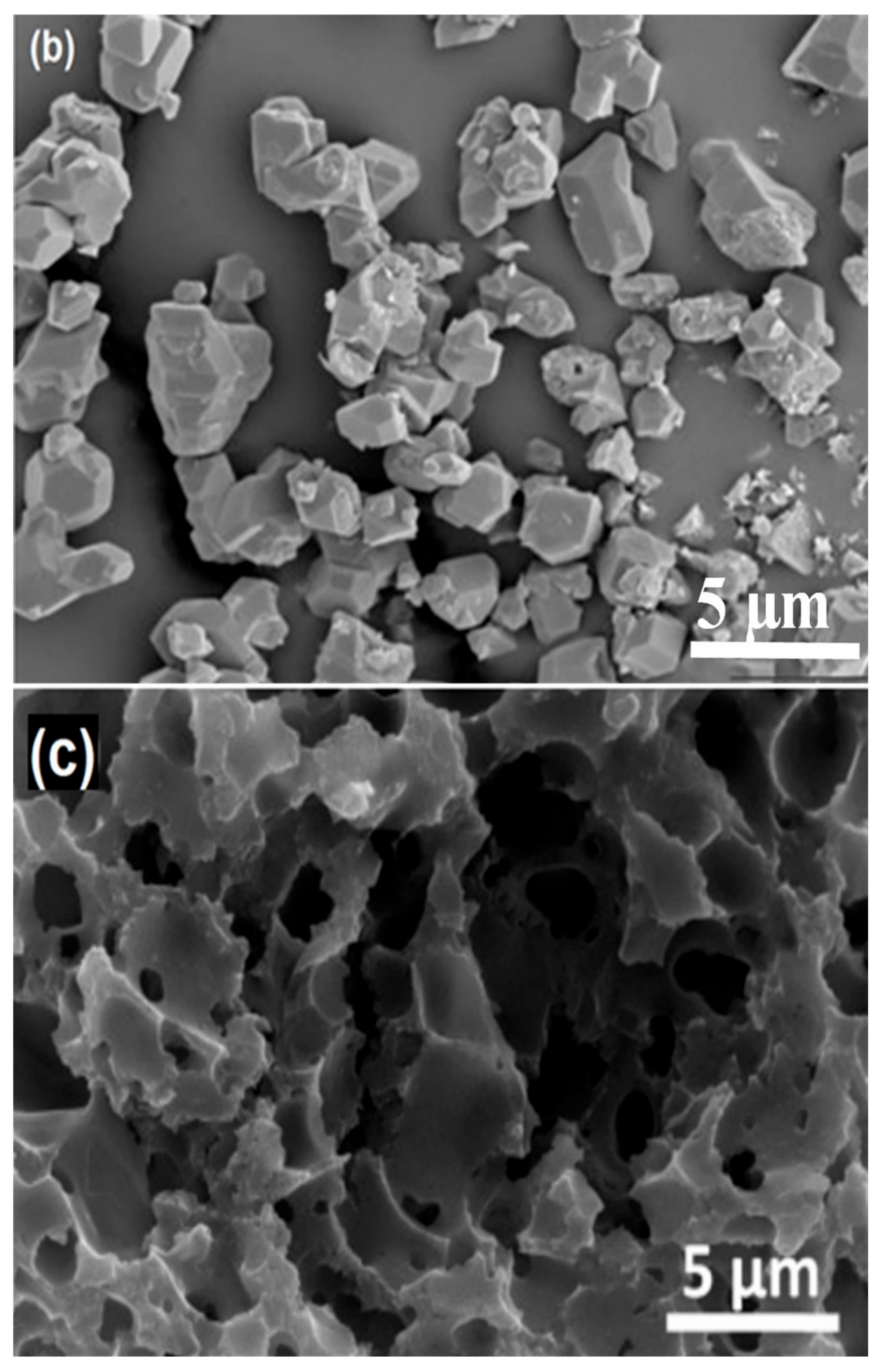
SEM of the CH (**a**), Zn@H_3_BTC (**b**), and CH/Zn@H_3_BTC (**c**).

FESEM was used to inspect the surface morphology of CH, Zn@H_3_BTC, and CH/Zn@H_3_BTC. The morphological features of CH, which has a smooth surface, are presented in Figure 3a. Figure 3b depicts a microscopic view of the geometry of Zn@H_3_BTC powder, which has an irregular polyhedral structure with a pore size range of 500–800 nm. Figure 3c shows the porous morphology of CH/Zn@H_3_BTC, which is characterized by micron-sized homogeneously dispersed spherical pores. The presence of chitosan should have aided the formation of smooth porous spheres. For CH/Zn@H_3_BTC, however, the morphology was altered to a more finely dispersed mesoporous structure. The structure of Zn@H_3_BTC remained constant in the absence of CH. The prepared CH/Zn@H_3_BTC ultimately condensed in the presence of CH due to CH’s cohesiveness. This showed that CH has a minimal influence on the morphology of Zn@H_3_BTC and that the surface of Zn@H_3_BTC was successfully capped with CH.

#### 3.1.4. BET Analysis

**Figure 4:**
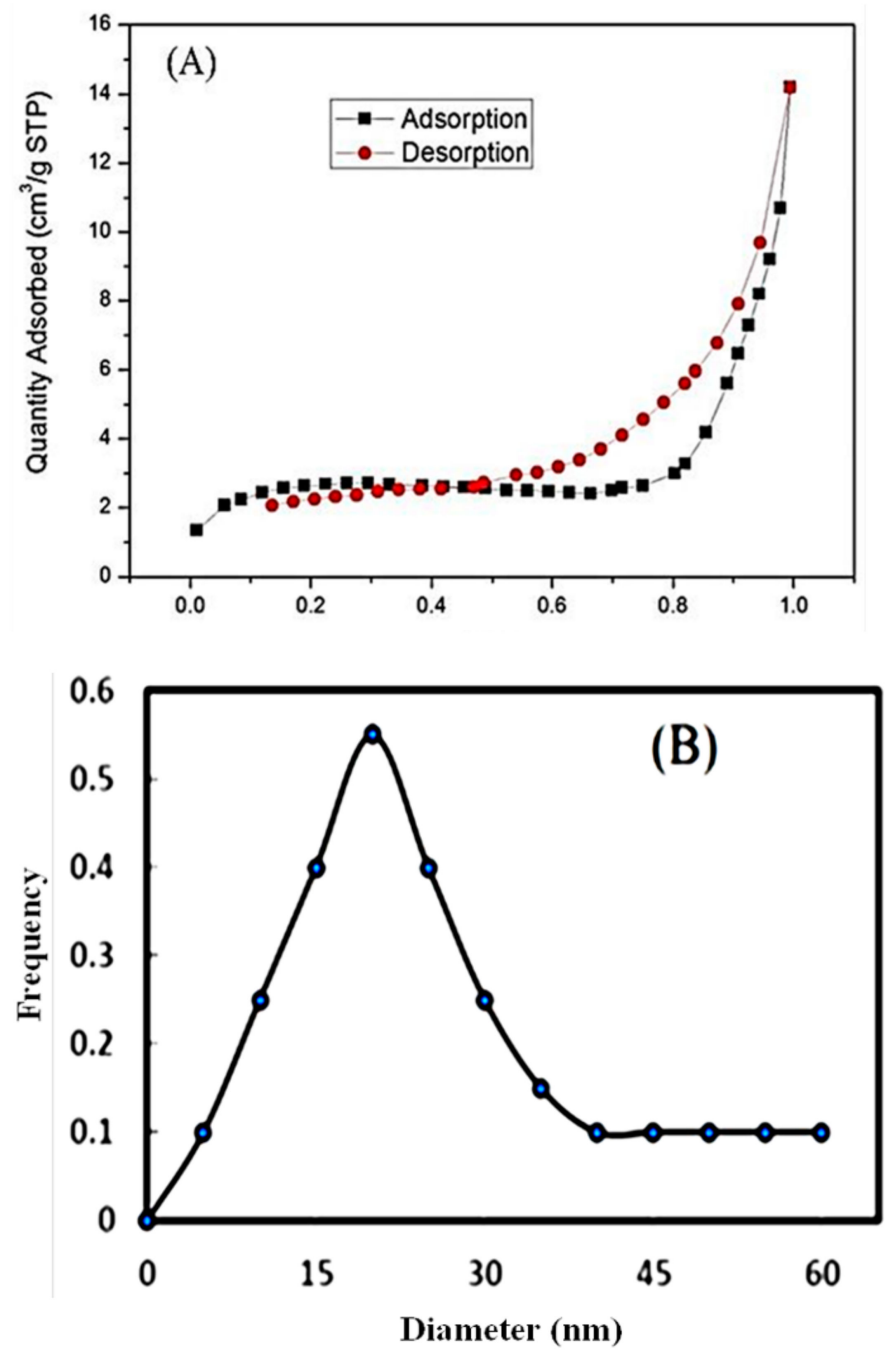
N_2_ adsorption-desorption isotherm at 77.35 K, for CH/Zn@H_3_BTC (**A**) and the pore size distribution of CH/Zn@H_3_BTC (**B**).

The N_2_ adsorption–desorption isotherms of CH/Zn@H_3_BTC are shown in Figure 4A. The results demonstrated that the first inflection point is at p/p0 < 2, which indicates the completion of monolayer adsorption. As the pressure increased, the adsorption of the second layers occurred. The number of adsorption layers is limitless at saturated vapor pressure so the adsorption isotherm is characteristic of type II. According to the multipoint BET technique, the surface area of CH/Zn@H_3_BTC was estimated as 80 m^2^/g. In addition to the pore size distribution of CH/Zn@H_3_BTC, Figure 4B displays a mean pore diameter of 22.5 nm, which confirmed the mesoporosity of the CH composite. This reasonable mesoporous feature would help the CH/Zn@H_3_BTC composite to easily diffuse and adsorb on steel substrates to hence act as a potential corrosion inhibitor.

### 3.2. Corrosion Protection Evaluation

#### 3.2.1. *E*_OCP_−*t* Profiles

**Figure 5:**
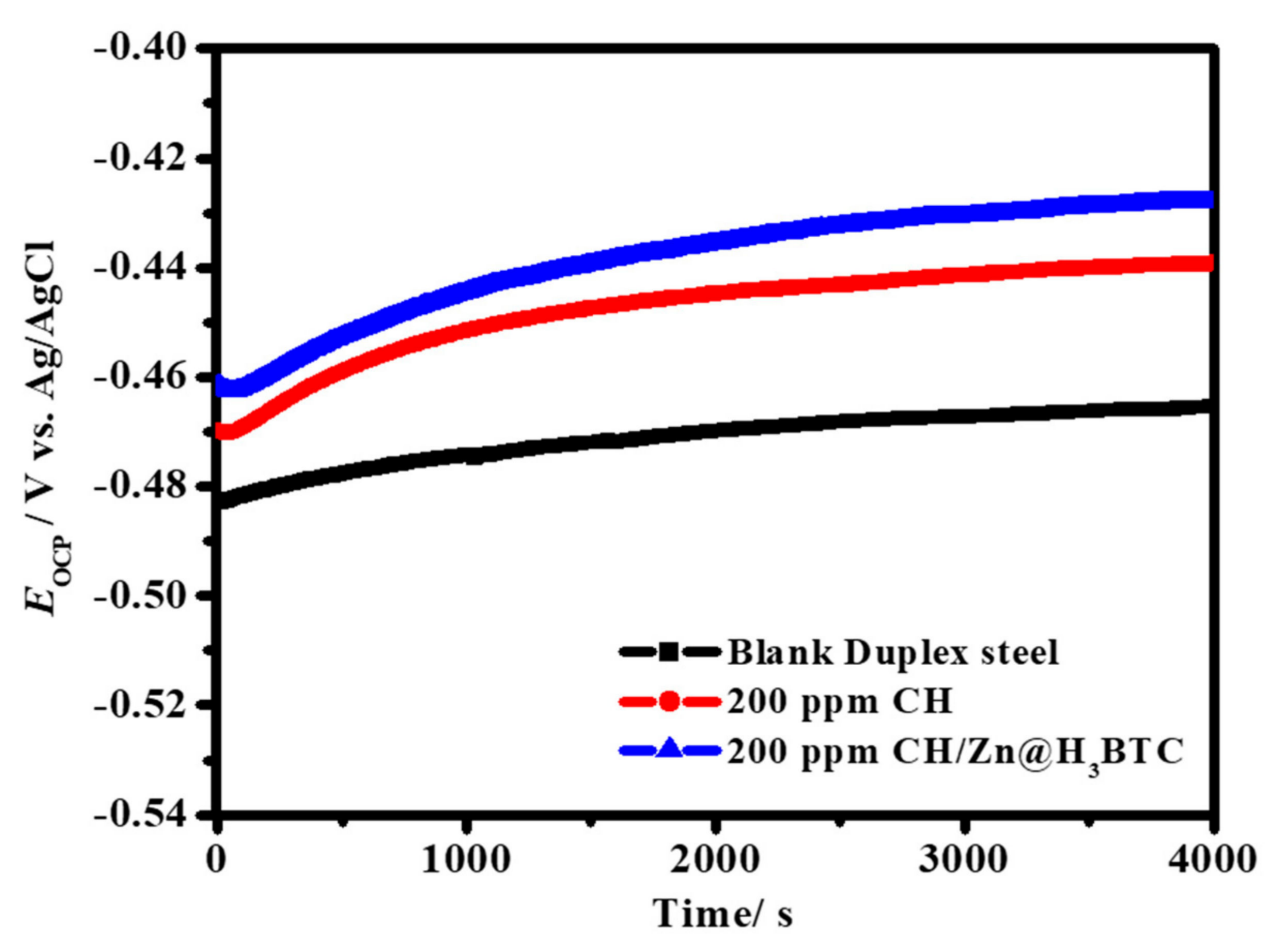
*E*_OCP_ versus t profiles for duplex steel in a 2.0 M sulfuric acid medium without and with 200 ppm CH and CH/Zn@H_3_BTC.

A steady potential lacking an external current was applied on the duplex steel’s interface for EIS and PP experiments. Figure 5 displays the profiles of *E*_OCP_ (vs. a Ag/AgCl electrode) in 2.0 M sulfuric acid medium without and with 200 ppm of CH and CH/Zn@H_3_BTC as a function of dipping time at 323 K. For the blank and solutions containing 200 ppm (optimum dose) of CH and CH/Zn@H_3_BTC, various immersion times were necessary to attain a stable *E*_OCP_ value. It can be observed from Figure 5 that 50 min was sufficient to attain a stable *E*_OCP_, so this time was consequently employed as the exposure time before PP and EIS experiments.

#### 3.2.2. PDP Studies and Comparative Evaluation

**Figure 6:**
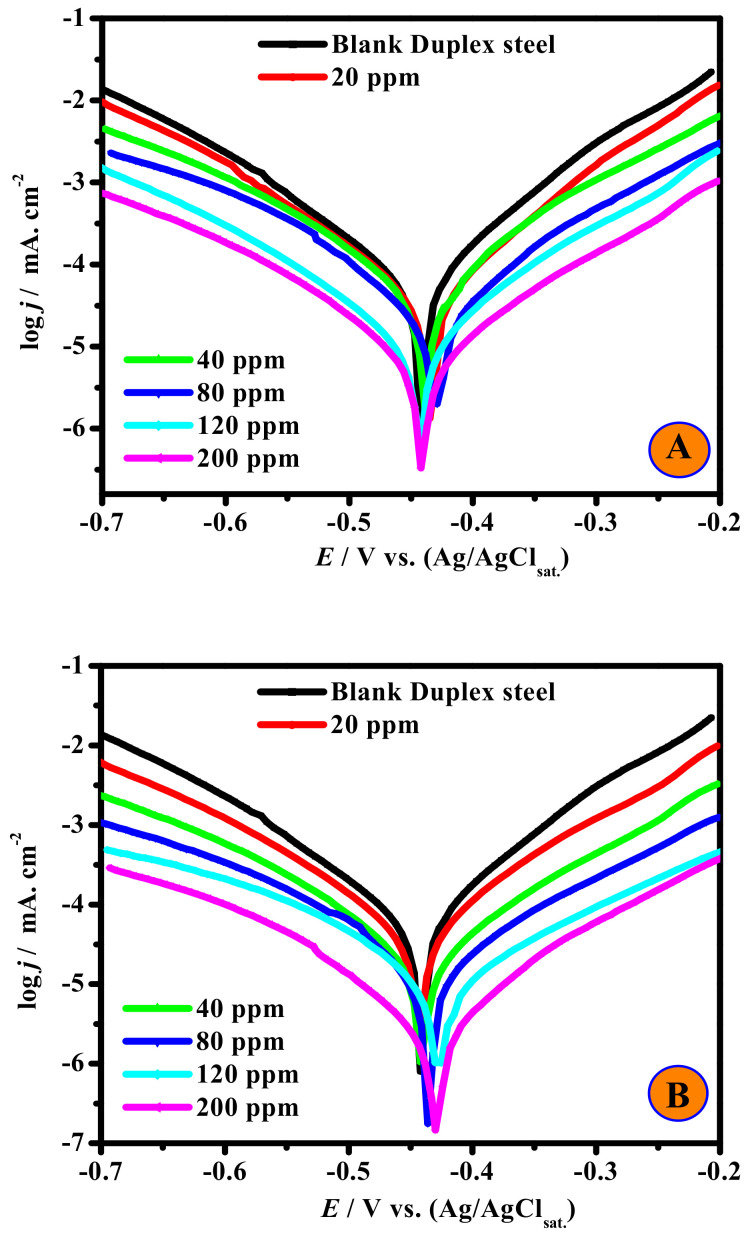
Tafel profiles for duplex steel at 323 K in 2.0 M H_2_SO_4_ without and with various concentrations of (**A**) CH and (**B**) CH/Zn@H_3_BTC.

Figure 6 presents the Tafel plots of duplex steel in 2.0 M sulfuric acid medium without and with various concentrations of CH (Figure 6A) and CH/Zn@H_3_BTC (Figure 6B) at 323 K. In Figure 6, anodic and cathodic Tafel lines move clearly to lower current values with the addition of CH and CH/Zn@H_3_BTC, due to the insertion of CH and CH/Zn@H_3_BTC in the corrosive solution inhibiting the hydrogen ions’ reduction and reducing the anodic dissolution of the metal. Furthermore, the decreases in the density of the cathode current values were more noticeable than those of the anode, which showed that the adsorption of CH and CH/Zn@H_3_BTC on the metal surface efficiently prevents hydrogen evolution at the cathode side.

To understand the mechanism of the inhibition process of CH and CH/Zn@H_3_BTC on duplex steel in 2.0 M H_2_SO_4_, the important PP parameters such as corrosion voltage (*E*_corr_), corrosion current density (*j*_cor_), and anodic and cathodic Tafel slopes (*β*_a_ and *β*_c_) are recorded in Table 1. By extrapolation of the anodic and cathodic lines, the *E*_cor_ and *j*_cor_ values were obtained. The inhibition capacity (*η*_pp_/%) is calculated by Equation (1) as follows [39]:

(1) $$\eta_{\mathrm{PP}}/\%=\left(\frac{j_{\mathrm{cor},\;o}-j_{\mathrm{cor},i}^{}}{j_{\mathrm{cor},\;o}}\right)\times 100=\theta \times 100$$

where *j*_cor,o_ and *j*_cor,i_ are *j*_cor_ with and without CH and CH/Zn@H_3_BTC in 2.0 M H_2_SO_4_, respectively.

Table 1 shows that the *β*_a_ and *β*_c_ values were different for the different concentrations CH and CH/Zn@H_3_BTC and the blank system, demonstrating that CH and CH/Zn@H_3_BTC can control the anode and cathode reactions [40]. Moreover, the change in the (Δ*E*_cor_) *E*_cor_ values was less than 0.085 V vs. Ag/AgCl in all cases, showing that CH and CH/Zn@H_3_BTC are mixed-kind inhibitors [41]. The *j*_cor_ value declined from 1137.7 µA cm^−2^ for the uninhibited system to 713.33 and 613.22 µA cm^−2^ with the addition of 20 ppm of CH and CH/Zn@H_3_BTC, respectively, which further diminished to 67.12 and 25.02 µA cm^−2^ for 200 ppm CH and CH/Zn@H_3_BTC, respectively. The protection ability of CH and CH/Zn@H_3_BTC, as calculated by Equation (1), are 37.3% and 46.1%, and 94.1% and 97.8% at concentrations of 20 and 200 ppm, respectively. By comparing the findings with those of previously reported compounds [42,43], we found the protection efficacy of CH and CH/Zn@H_3_BTC is clearly stronger. The improved protection capability might be attributed to the bonded functional chitosan and benzene ring owing to the extra donor–acceptor attractions among the empty d orbitals of iron atoms and the π electrons.

#### 3.2.3. EIS Studies

**Figure 7:**
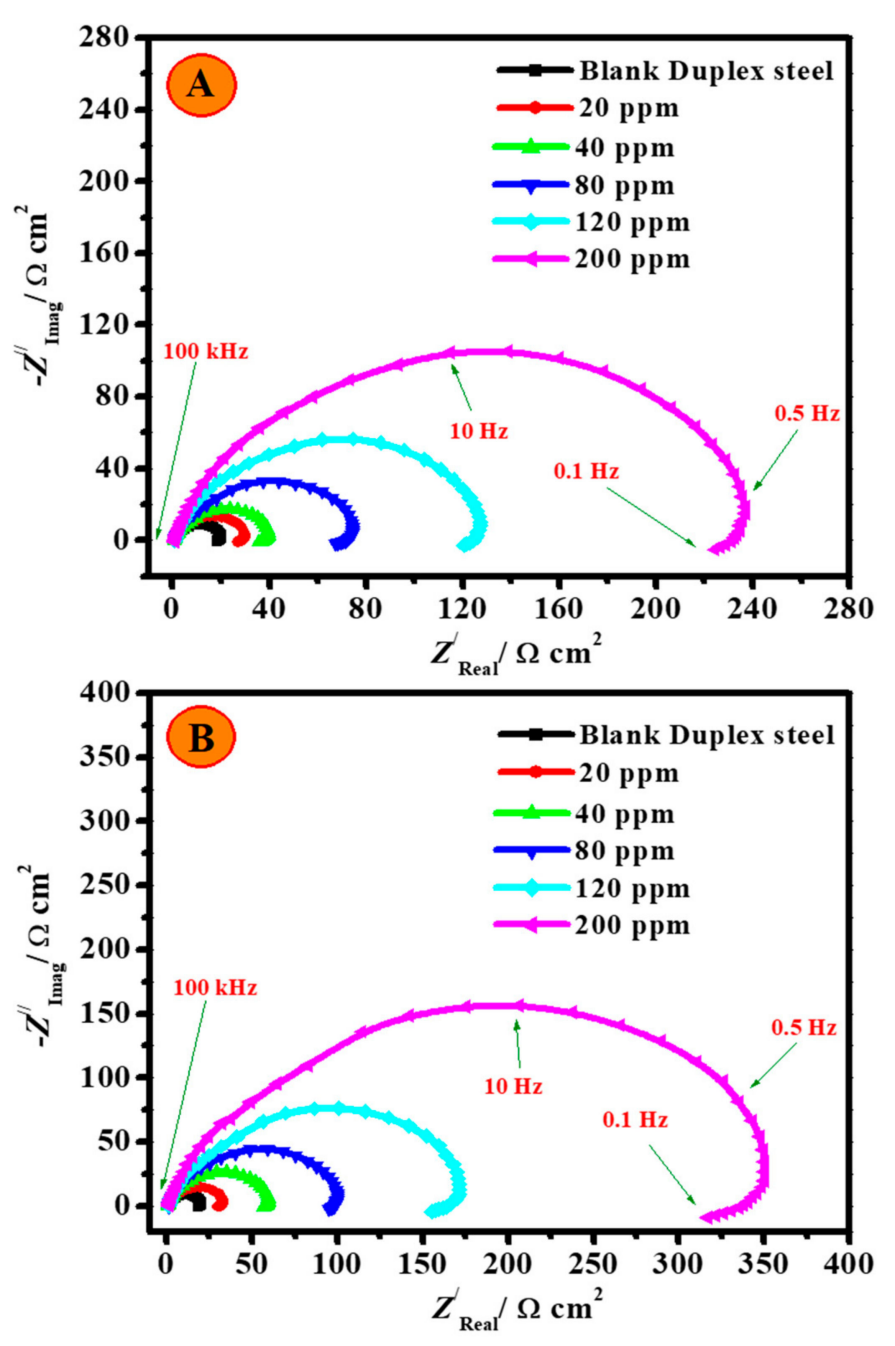
Nyquist plots for duplex steel at 323 K in 2.0 M H_2_SO_4_ with and without various concentrations of (**A**) CH and (**B**) CH/Zn@H_3_BTC.

The corrosion performance of duplex steel in a 2.0 molar sulfuric acid medium both with and without the prepared CH and CH/Zn@H_3_BTC composite was examined by EIS at 50 ± 1 °C at *E*_OCP_. The kinetics of the electrode, surface characteristics, and mechanism were attained from the EIS graphs [44]. The Nyquist plots of duplex steel in the investigated corrosive medium at 323 K without and with various doses of CH polymer and CH/Zn@H_3_BTC composite are depicted in Figure 7A,B. We observed that the Nyquist profiles were imperfect capacitive loops (Figure 7), which showed that the metal dissolution was controlled by the charge shift route in the studied corrosive medium [45]. The capacitive loop (Figure 7) displayed a nonperfect arc in a 2.0 M H_2_SO_4_ medium owing to the inhomogeneity and roughness of the duplex steel interface.

After investigating the Nyquist profile shape, we found that the Nyquist diagrams exhibited a depressed capacitive loop in the higher-frequency (HF) area and a minor inductive loop in the lower-frequency (LF) area, demonstrating the existence of a Faradic process on the permitted metal sites. The capacitive loop in the HF area was recognized as time-continuous for the double electric layer and the charge transfer process. However, the inductive loop at the LF area was ascribed to the recreation of intermediates regulating the corrosion process due to species adsorption, for instance, FeSO_4_ [46], or inhibitor molecules [47] on the metal interface. It may also be related to the re-dissolution of the oxide film in the lower-frequency range [48]. The capacitive loop diameter with the CH polymer and CH/Zn@H_3_BTC composite was larger than that of the blank system, and increased with increasing dose. This showed that the resistance of the inhibited steel surface increased with the inhibitor concentration. The diameter of the semi-circle followed the order of CH/Zn@H_3_BTC composite < CH polymer under the same conditions, which showed that the most active adsorption film on the electrode interface was produced with CH/Zn@H_3_BTC composite in 2.0 M sulfuric acid medium, which powerfully obstructed the effects of corrosion of the acid medium.

**Figure 8:**
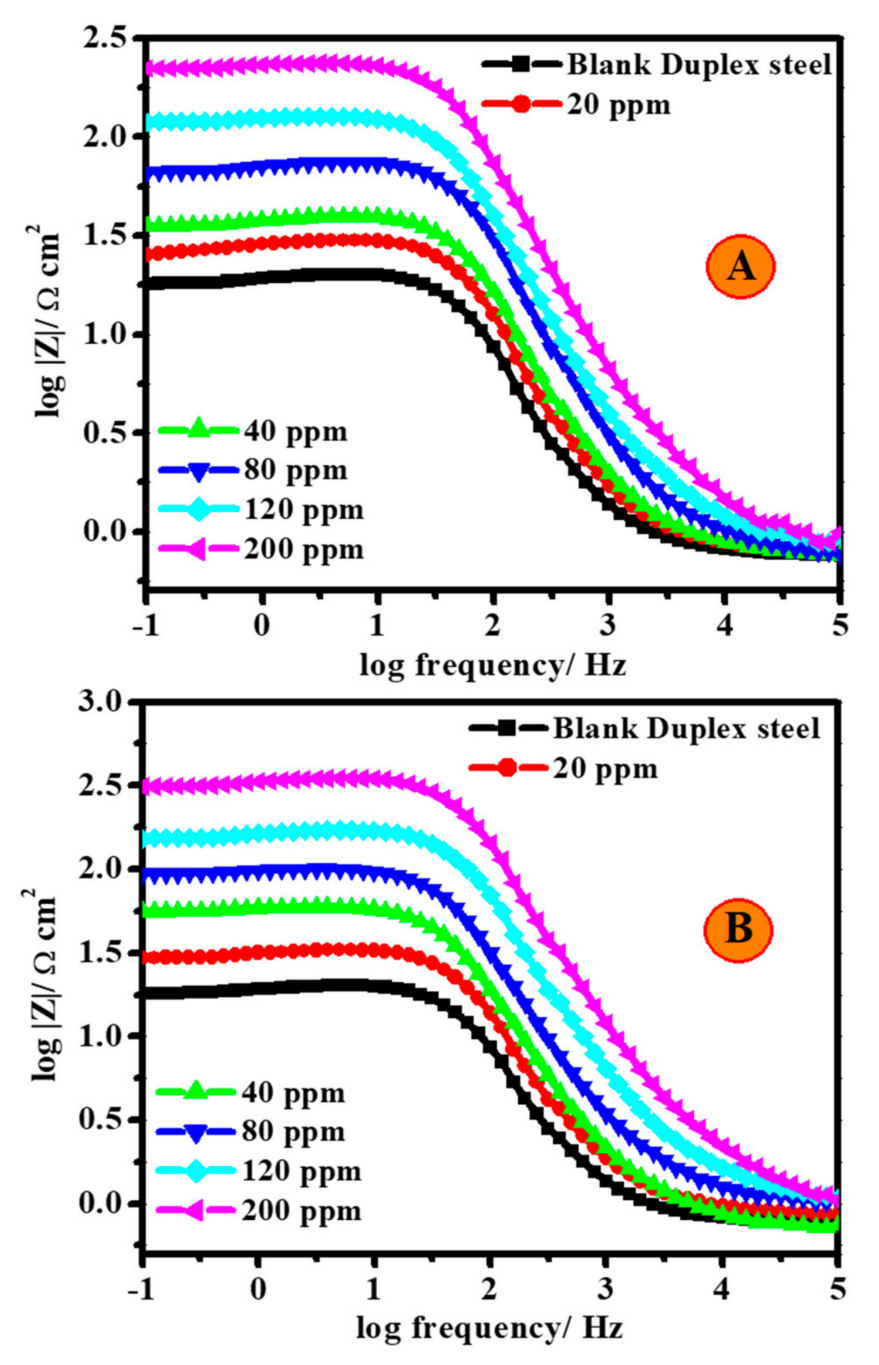
Bode profiles for duplex steel at 323 K in 2.0 M H_2_SO_4_ with and without various concentrations of (**A**) CH and (**B**) CH/Zn@H_3_BTC.

**Figure 9:**
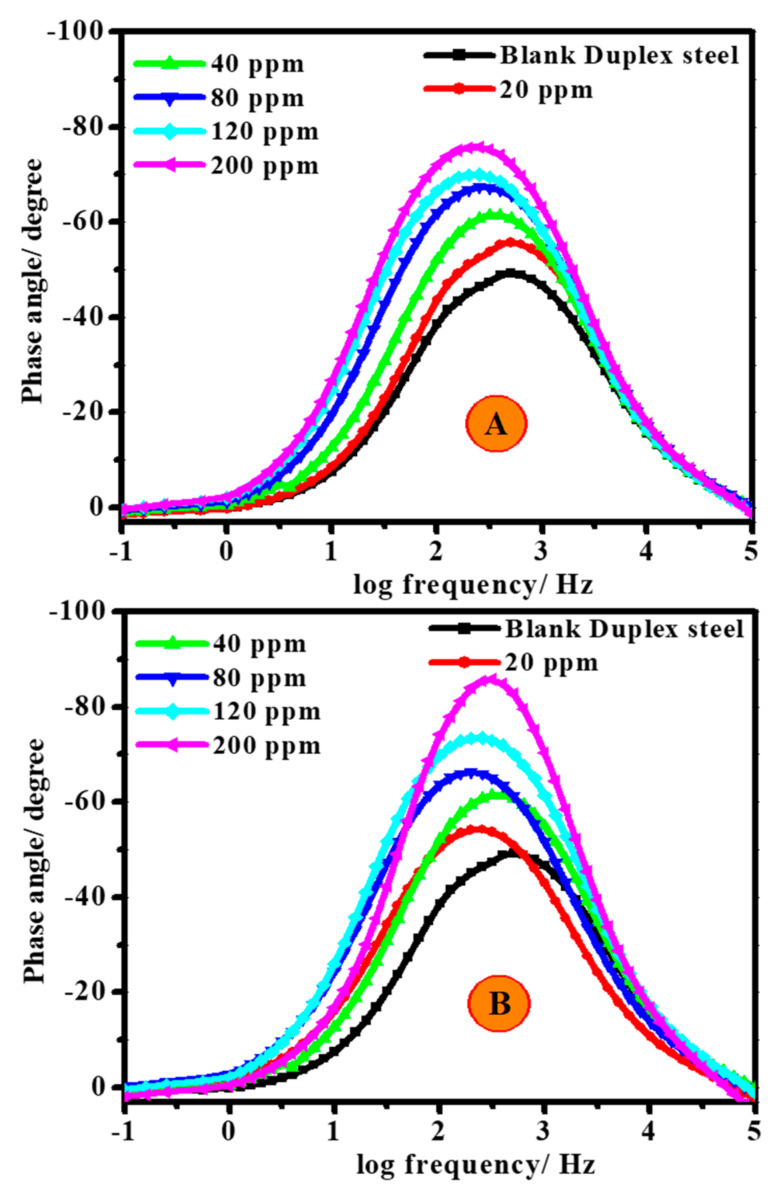
Bode phase profiles for duplex steel at 323 K in 2.0 M H_2_SO_4_ with and without various concentrations of (**A**) CH and (**B**) CH/Zn@H_3_BTC.

Bode profiles for duplex steel at 323 K in 2.0 M H_2_SO_4_ without and with various concentrations of CH and CH/Zn@H_3_BTC are presented in Figure 8 and Figure 9. A single time constant was detected in the Bode profiles, demonstrating that the duplex steel corrosion in the studied corrosive medium was predominantly organized by a charge transfer route. At the electrode/medium interface, the charge sharing on the electrode cross was well-ordered by electrons; however, it was orderly by the ions on the medium side. Subsequently, there were many more ions than electrons. A slight difference in capacitance was observed compared to a perfect capacitor at the steel/electrolyte interface [49]. As revealed in Figure 8 and Figure 9, the phase angle and impedance modulus values considerably increased with increasing doses of CH and CH/Zn@H_3_BTC. The impedance modulus value increased by two orders of magnitude and the phase angle increased to 82° when the optimum dose of CH/Zn@H_3_BTC was used (200 ppm), which showed that CH/Zn@H_3_BTC adsorption efficiently impedes the corrosion of duplex steel in 2.0 M H_2_SO_4_ [50].

**Figure 10:**
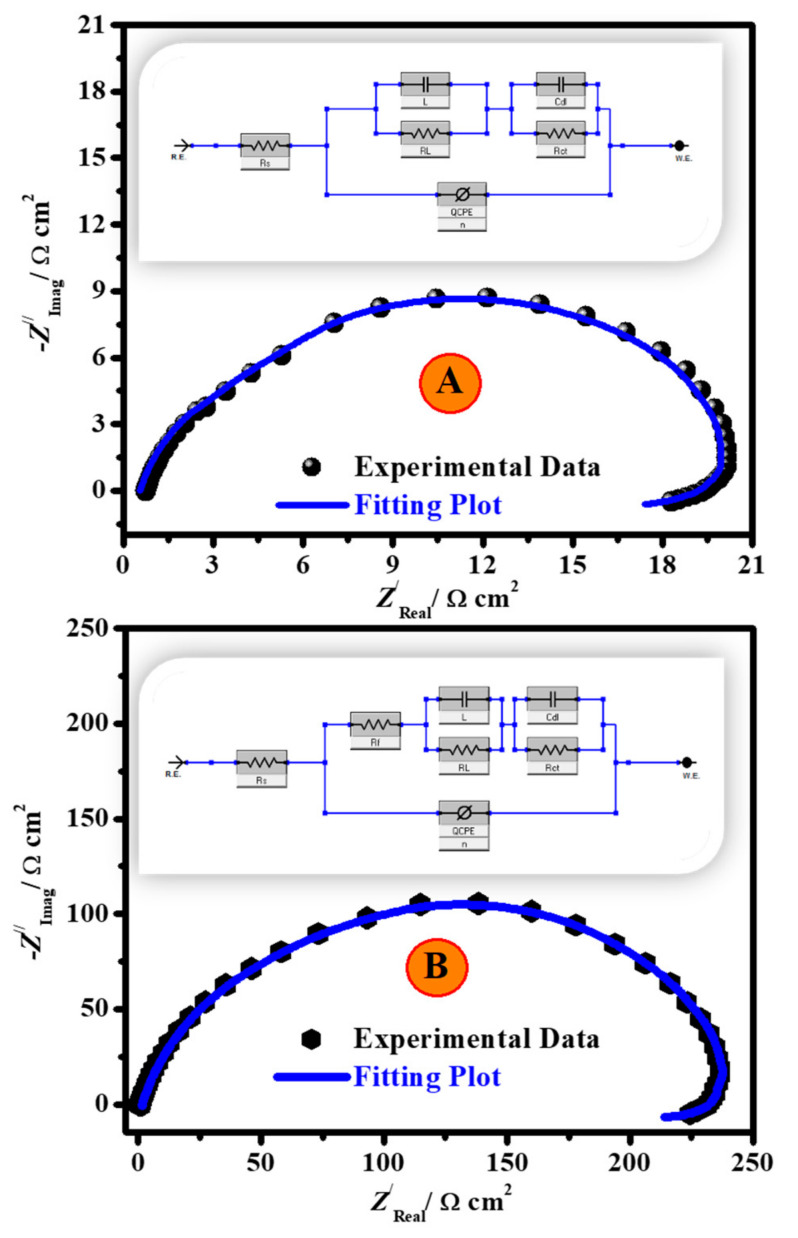
Nyquist profiles and fitted plots or duplex steel in blank 2.0 M H_2_SO_4_ solution (**A**) and in the presence of 200 ppm of CH (**B**) at 50 °C. Inset, equivalent circuit used to fit the EIS findings.

Nyquist profiles and fitted plots (—) for duplex steel in blank 2.0 M H_2_SO_4_ solution and in the presence of 200 ppm of CH at 50 °C are presented in Figure 10A,B, respectively, and an electrical equivalent circuit (EEC) is presented in the Figure 10 inset. The EEC was used to measure the electrolyte impedance (*R*_s_), the resistance of charge transfer (*R*_ct_), the constant phase element (*CPE*), the inductance resistor (*R*_L_), and the inductance (*L*), in addition to the film resistance (*R*_f_) in the case of the inhibited system. The CPE impedance is computed through the following formula [51]:

(2) $$Z_{CPE}^{}=\frac{1}{Y_{0}{\left(j\omega \right)}^{n}}$$

where *Y*_0_ is the *CPE* constant, *j* is the $\sqrt{i}$, *ω* is the angular frequency, and *n* is the deviation factor. The capacitance of double layer (*C_dl_*) was utilized rather than CPE because it could more precisely fit the impedance parameters. The *C_dl_* value was calculated by the Brug formula [52]:

(3) $$C_{dl}=Y_{0}^{\frac{1}{n}}{\left(\frac{1}{R_{s}}+\frac{1}{R_{\mathrm{ct}}}\right)}^{1-\frac{1}{n}}$$

The protection ability *(η*%) was computed from the resistance of the charge transfer (*R*_ct_) values as follows [53]:

(4) $$\eta /\%=\left(\frac{R_{\mathrm{ct}}^{i}-R_{\mathrm{ct}}^{f}}{R_{\mathrm{ct}}^{i}}\right)\times 100$$

where $R_{\mathrm{ct}}^{i}$ and $R_{\mathrm{ct}}^{f}$ represent the *R*_ct_ for the free and inhibited systems, respectively. The fit of the EEC was evaluated by the chi-square (*χ*^2^) value [53]. The attained *χ*^2^ values (3.91 × 10^−4^ to 5.48 × 10^−4^) in Table 2 show an appropriate fit to the EEC. The *R*_ct_ value increased from 20.6 to 30.4 and 33.3 Ω cm^2^ when the CH and CH/Zn@H_3_BTC doses reached 20 ppm, respectively. The values of *R*_ct_ reached the highest values at 252.4 and 364.8 6 Ω cm^2^ at 200 ppm for CH and CH/Zn@H_3_BTC, respectively, demonstrating the decline in the rate of steel corrosion. Consequently, *η*% values of 91.8% and 94.3% were obtained for CH and CH/Zn@H_3_BTC, respectively, demonstrating the promising corrosion protection ability of CH polymer and CH/Zn@H_3_BTC composite for duplex steel in the investigated corrosive medium. Due to the appropriate CH polymer and CH/Zn@H_3_BTC composite adsorption at the electrode/electrolyte interface, the pre-adsorbed H_2_O molecules were increasingly replaced by the inhibitor components with a small dielectric constant. Accordingly, *C_dl_* decreased with the increase in CH polymer and CH/Zn@H_3_BTC composite dose according to the subsequent Equation (5):

(5) $$C_{dl}=\left(\frac{\varepsilon \varepsilon_{0}}{d}\right)\times S$$

where *ε*_0_ and *ε* are the constants of a vacuum and the local dielectric, respectively; *d* represents the double film thickness; and *S* is the active electrode area. The adsorption of CH polymer and CH/Zn@H_3_BTC composite on the duplex steel interface decreased ε and increased *d*. Consequently, *C_dl_* was lower for the duplex steel in the inhibited system. The continual increase in *R*_ct_ and the decrease in *C_dl_* also showed that the adsorption film of CH polymer and CH/Zn@H_3_BTC composite was inclined to be compact and uniform. Based on their adsorption, the value of *n* regularly increased and nearly reached unity. Higher *n* values were detected for the sample in the acidic solution containing CH polymer and CH/Zn@H_3_BTC inhibitors. This indicated a comparatively homogeneous and smooth surface owing to the inhibitor adsorption. In summary, the EIS differences in the protection competencies agree with that achieved from the PP method.

### 3.3. Adsorption Considerations, Thermodynamic Research, and Corrosion Mitigation Mechanism

Corrosion mitigation properties were attained through to the adsorption of inhibitor species onto the electrode interface to form a compact defensive layer and protect the electrode from the acidic solution. Alternatively, the adsorbed inhibitor species may integrate with the oxide film on the steel (rust) and chemically interact to yield an additional protecting interface system by altering the construction of the layer. The adsorption processes of inhibitor molecule occur directly based on donor–acceptor attractions among the comparatively insecure bound electrons, for instance, in anions, inhibitor molecules, and/or the polymeric materials that have π electrons, or free coupled electrons with unoccupied d orbitals of the Fe atoms on the metallic interface. Some factors, including the size of the inhibitor molecule, the number of efficient groups, the polarity that influences the development of a robust bond, or the adsorption rate of inhibitor molecules onto a metallic interface, can disturb the inhibition and/or adsorption processes [54].

**Figure 11:**
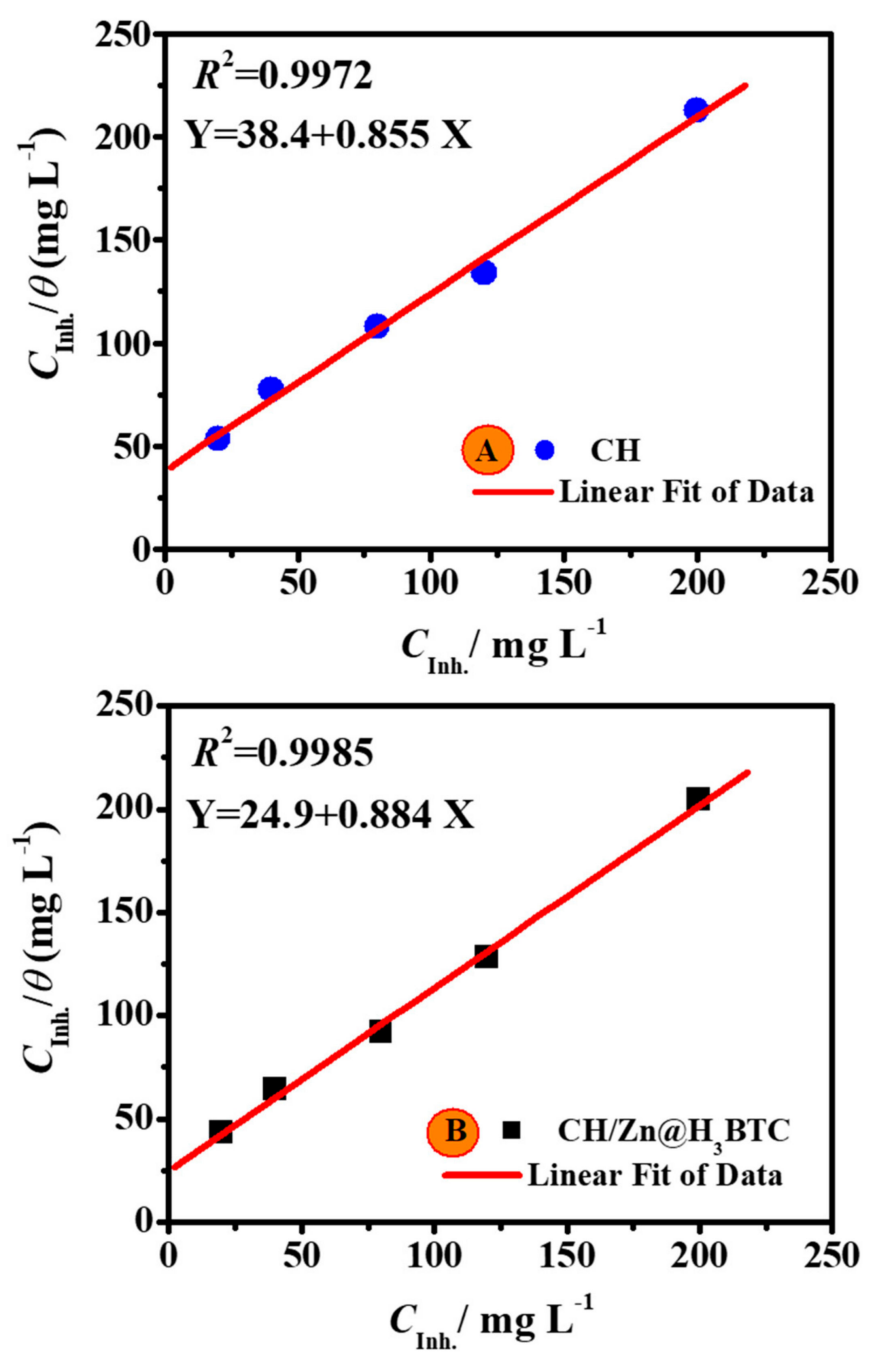
Plots of the Langmuir isotherm extracted from Tafel plots at 323 K in 2.0 M H_2_SO_4_ with and without various concentrations of (**A**) CH and (**B**) CH/Zn@H_3_BTC.

Some isotherm adsorption models, such as Frumkin, Temkin, Langmuir, El-Awady Flory–Huggins, and so on, were applied to fit PP the findings. These data showed that the Langmuir model displayed a respectable linear association with the experimental PP results. The isotherm of the Langmuir adsorption model is defined as follows [55]:

(6) $$\frac{C_{in}}{\theta }=\frac{1}{K_{ad}}+C_{in}$$

where *C_in_* is the inhibitor dose, *K_ad_* is the constant of adsorptive stability, and *θ* is the part of covered surface. *C_in_*/*θ* versus *C_in_* produced a straight line with the regression coefficient (*R*^2^ = 0.9972 and 0.9985 for CH and CH/Zn@H_3_BTC, respectively) and the slope was close to unity (*S* = 0.855 and 0.884 for CH and CH/Zn@H_3_BTC, respectively) at the investigated temperature, as revealed in Figure 11. These findings demonstrated that the adsorption of CH and CH/Zn@H_3_BTC on metal surfaces is agreement with the Langmuir isotherm model. The free energy of standard adsorption ($\Delta G_{ads}^{0}$) is attained by the subsequent equation [55]:

(7) $$\Delta G_{ads}^{0}=-2.303RT\log(1\times 10^{6}K_{ads})$$

where *R* represents the universal gas constant, *T* is the absolute temperature, and 1 × 10^6^ is the H_2_O concentration. The greater the *K_ad_* value (*K_ad_* = 0.026 and 0.041 L/g for CH and CH/Zn@H_3_BTC, respectively), the larger the area of covered surface, and the better the anticorrosion film of CH and CH/Zn@H_3_BTC on the electrode interface. The computed values of $G_{ads}^{0}$ were found to be −27.3 and −28.5 kJ g^−1^ for CH and CH/Zn@H_3_BTC, respectively. The negative values of $G_{ads}^{0}$ suggested the spontaneous adsorption of CH and CH/Zn@H_3_BTC at the electrode/electrolyte interface [56].

In this study, the $G_{ads}^{0}$ values were found to be greater than −20 kJ g^−1^ and smaller than −40 kJ g^−1^, showing that the mechanism of adsorption of the CH and CH/Zn@H_3_BTC on duplex steel in 2.0 M sulfuric medium was mainly based on chemical and physical adsorption [57].

Consequently, physisorption might occur owing to electrostatic attractions among the protonated inhibitor molecule and ${\mathrm{FeSO}}_{4}^{2-}$ species. The chemical adsorption of the CH polymer and CH/Zn@H_3_BTC composite on the metallic interface might occur through donor–acceptor attractions between the π electrons of O (and/or N) fragments and the empty d orbitals of iron atoms. Furthermore, with the attraction of the chitosan chain to the metal interface, polymer long chains correspondingly play a significant role in the inhibition and adsorption performance.

The mechanism in acidic media comprising two adsorbed intermediates was suggested for the hindrance of iron anodic dissolution in the presence of an inhibitor, as follows [58]:

(8) $$\mathrm{Fe}+H_{2}O\;\underset{}{↔}\;\mathrm{Fe}.H_{2}O{\mathrm{ads}}_{\;}$$

(9) $$\mathrm{Fe}.H_{2}O_{\mathrm{ads}}+\mathrm{Inh}.\;\underset{}{↔}\;\mathrm{Fe}.H_{2}{O^{-}}_{\mathrm{ads}}+H^{+}+\mathrm{Inh}.$$

(10) $$\mathrm{Fe}.H_{2}O_{\mathrm{ads}}+\mathrm{Inh}.\;\underset{}{↔}\;\mathrm{Fe}.\mathrm{Inh}._{\mathrm{ads}}+H_{2}O$$

(11) $$\mathrm{Fe}.\mathrm{OH}{}_{\mathrm{ads}}^{-}+\mathrm{Inh}.\;\overset{rds}{\to }\;{\mathrm{FeOH}}_{\mathrm{ads}}+e\;$$

(12) $$\mathrm{Fe}.\mathrm{Inh}._{\mathrm{ads}}.\;\underset{}{↔}\;\mathrm{Fe}.\mathrm{Inh}._{\mathrm{ads}}+e$$

(13) $${\mathrm{FeOH}}_{\mathrm{ads}}.+\mathrm{Fe}.\mathrm{Inh}._{\mathrm{ads}}\underset{}{↔}\;\mathrm{Fe}.\mathrm{Inh}._{\mathrm{ads}}+{\mathrm{FeOH}}^{+}$$

(14) $${\mathrm{FeOH}}^{+}+H^{+}\underset{}{↔}\;\mathrm{Fe}2^{+}+H_{2}O\;$$

where Inh. Represents the inhibitor molecule.

### 3.4. Surface Topography Exploration

**Figure 12:**
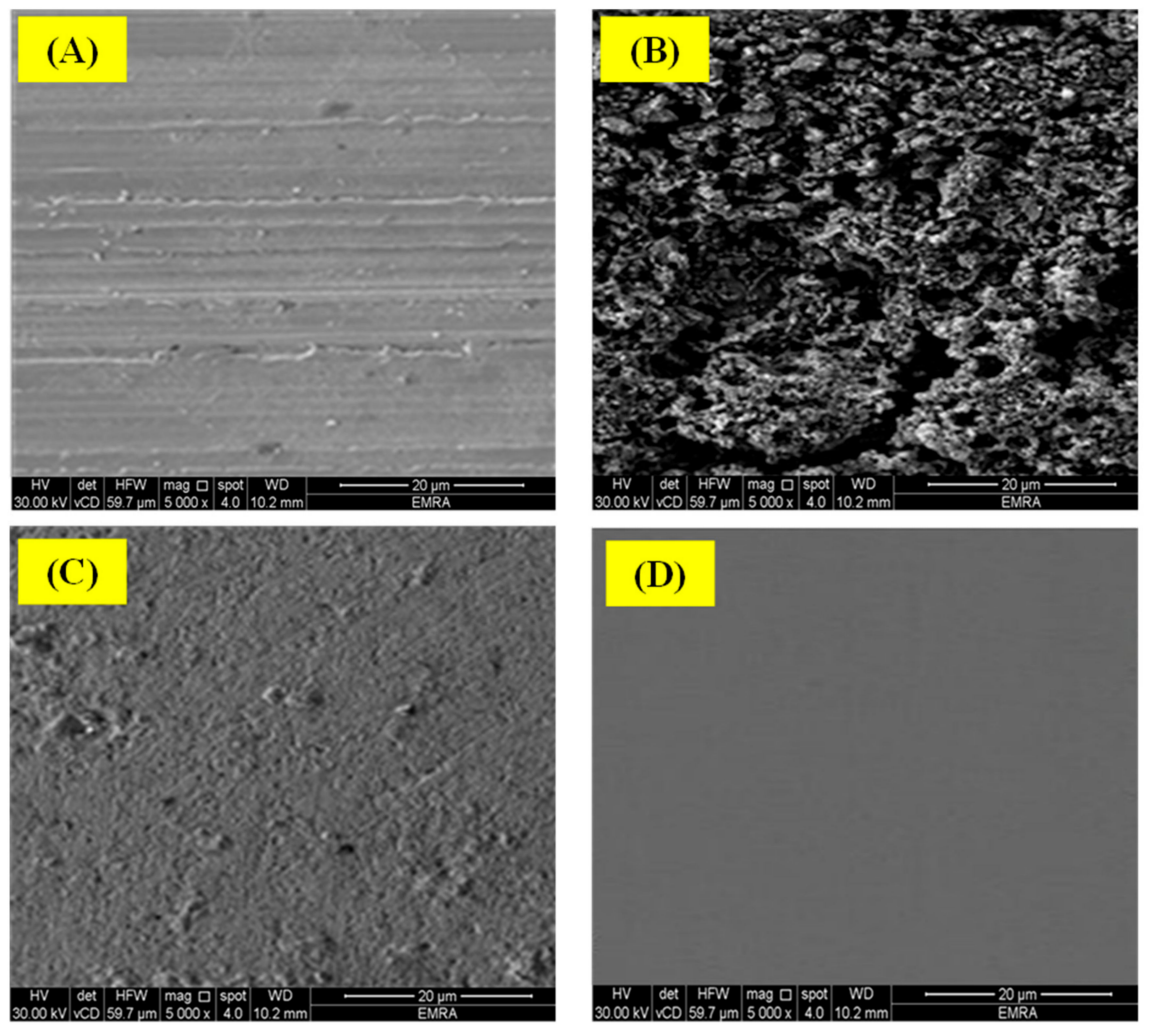
FE-SEM images of the pristine duplex steel (**A**) substrates after 24 h dipping in blank 2.0 M H_2_SO_4_ solutions without (**B**) and with of 200 ppm CH polymer (**C**) and CH/Zn@H_3_BTC composite (**D**).

In order to understand the inhibition characteristics of the CH polymer and CH/Zn@H_3_BTC composite, the surface topology of duplex steel was investigated. The FE-SEM images of the electrode interface after 24 h of submersion in 2.0 M sulfuric acid both with and without 200 ppm of CH polymer and CH/Zn@H_3_BTC composite are presented in Figure 12A–D. We observed big pits and deep cracks on the electrode surface (Figure 12B) in the solution without inhibitors, which was significantly different from the pristine metal, as shown in Figure 12A. This showed that duplex steel extremely corroded in the 2.0 M sulfuric medium. However, the steel surface showed a comparatively smoother surface morphology with 200 ppm CH polymer and CH/Zn@H_3_BTC composite, which was comparable to the pristine surface (Figure 12C,D). In comparison, in the presence of 200 ppm CH/Zn@H_3_BTC composite (Figure 12D), the metal interface displayed much less corrosion, i.e., a much smoother surface. Furthermore, these findings showed that the CH polymer and CH/Zn@H_3_BTC composite adsorbed on the electrode surface to produce a defensive layer that prohibited corrosion by the acidic solution. Therefore, the electrode was well-shielded by the CH polymer and CH/Zn@H_3_BTC composite in the studied acidic medium.

### 3.5. DFT Studies

**Figure 13:**
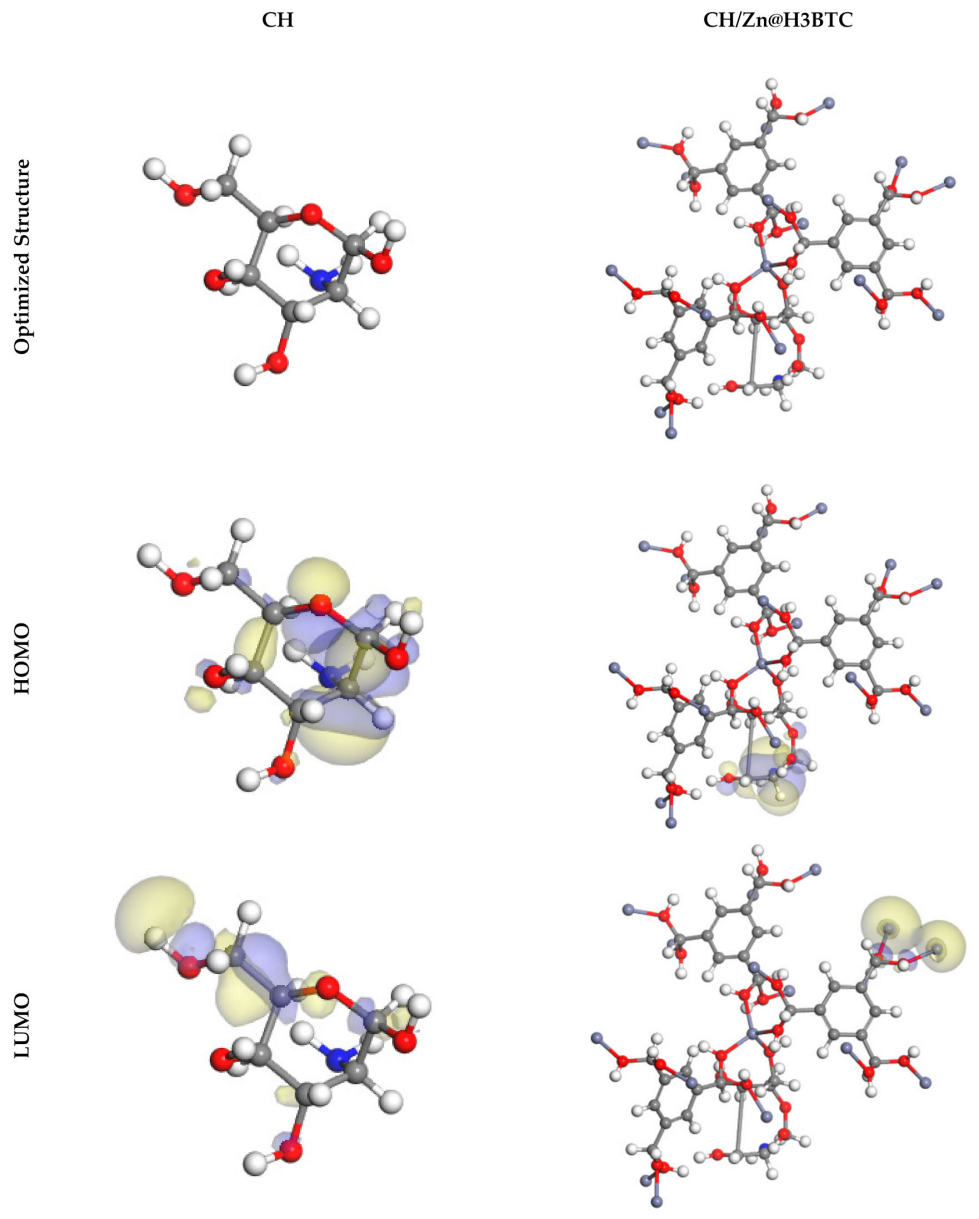
The molecular structures, HOMO, and LUMO of the monomer structures of CH and CH/Zn@H_3_BTC optimized using the DMol3 module.

DFT computations were performed to investigate the interaction between the active centers on CH and CH/Zn@H_3_BTC molecules and steel surfaces. The optimized constructions, HOMO and LUMO orbitals, for CH and CH/Zn@H_3_BTC molecules are shown in Figure 13, and the quantum chemical variables are listed in Table 3. Based on the FMO hypothesis, the provider and receiver capability of the inhibitor molecule in the inhibitor/metal exterior interaction are assigned by HOMO and LUMO energies [59]. In this way, a molecule that has large *E*_HOMO_ and low *E*_LUMO_ values is deemed to be an efficient corrosion inhibitor. As presented in Table 3, CH/Zn@H_3_BTC had a higher *E*_HOMO_ value (−9.11 eV) than CH (−5.00 eV). As presented in Figure 13, for inhibitor molecules, it is obvious that the HOMO level was situated on the N and O atoms; thus, these positions were chosen for the electrophilic study on the steel exterior. Such justifications approve the ability of CH and CH/Zn@H_3_BTC to be adsorbed on the steel exterior, thus demonstrating the anticorrosion ability, in agreement with the experimental outcomes. Moreover, the *E*_LUMO_ value was −8.57 eV for CH/Zn@H_3_BTC (Table 3), which is less than that of CH (0.81 eV), in agreement with experimental data; however, CH/Zn@H_3_BTC showed greater inhibition potency than CH. Additionally, the energy difference (Δ*E*) is a vital indicator of the prohibition ability of molecules, i.e., it increases as the Δ*E* value decreases [60]. As demonstrated in Table 3, CH/Zn@H_3_BTC had a lower Δ*E* value (0.55 eV) than CH (5.80 eV), which indicates the greater tendency of CH/Zn@H_3_BTC to be adsorbed on steel’s exterior.

Most common inhibitors demonstrate relatively low electronegativity (χ) values, indicating their ability to offer electrons to a metal surface [61]. In contrast, high electronegativity (χ) values indicate the strong ability of the inhibitor molecule to attract and take electrons from metal surface atoms (i.e., back-donation) and form a stronger bond with metal atoms [62]. From the computed outcomes listed in Table 3, we found that the electronegativity of CH/Zn@H_3_BTC is greater than that of CH. Consequently, CH/Zn@H_3_BTC demonstrated superior electron admission ability compared to CH [63]. Additionally, the stability and susceptibility of a compound can be evaluated from hardness (*η*) and softness (*σ*) values, i.e., soft compounds have stronger protection ability than hard compounds due to the integrity created by the smooth coating of electrons on the steel exterior during the adsorption, thus indicating efficient protective inhibitors [52]. As shown in Table 3, CH/Zn@H_3_BTC tended to have lower η values and higher σ values than CH, showing the strong ability to inhibit the donation of electrons to the examined steel, resulting in a strong anticorrosion ability.

The dipole moment is a vital indicator used to predict the prohibition effectiveness of a material [64]. The increase in dipole moment produced increases in deformation energy and molecule adsorption on the examined steel. Thus, the increases in dipole moment produced an increase in the corrosion inhibition potency [65]. As shown in Table 3, CH/Zn@H_3_BTC had a greater dipole moment value (53.16 Debye) than CH (6.09 Debye), which verified the strong tendency for CH/Zn@H_3_BTC to be adsorbed on steel and enhance the prohibition efficiency.

The ability of the examined particles to preserve the steel surface in the corrosive medium is related to their molecular surface area. The corrosion prohibition effectiveness increased as the molecular configuration increased because of the area over which the inhibitor molecules interacted with the steel surface increased. As shown in Table 3, CH/Zn@H_3_BTC had the largest molecular surface area, which supports the experimental data; the corrosion prohibition potency was higher for CH/Zn@H_3_BTC (1188.75 ${Å}^{2}$) than for CH (193.50 ${Å}^{2}$).

**Figure 14:**
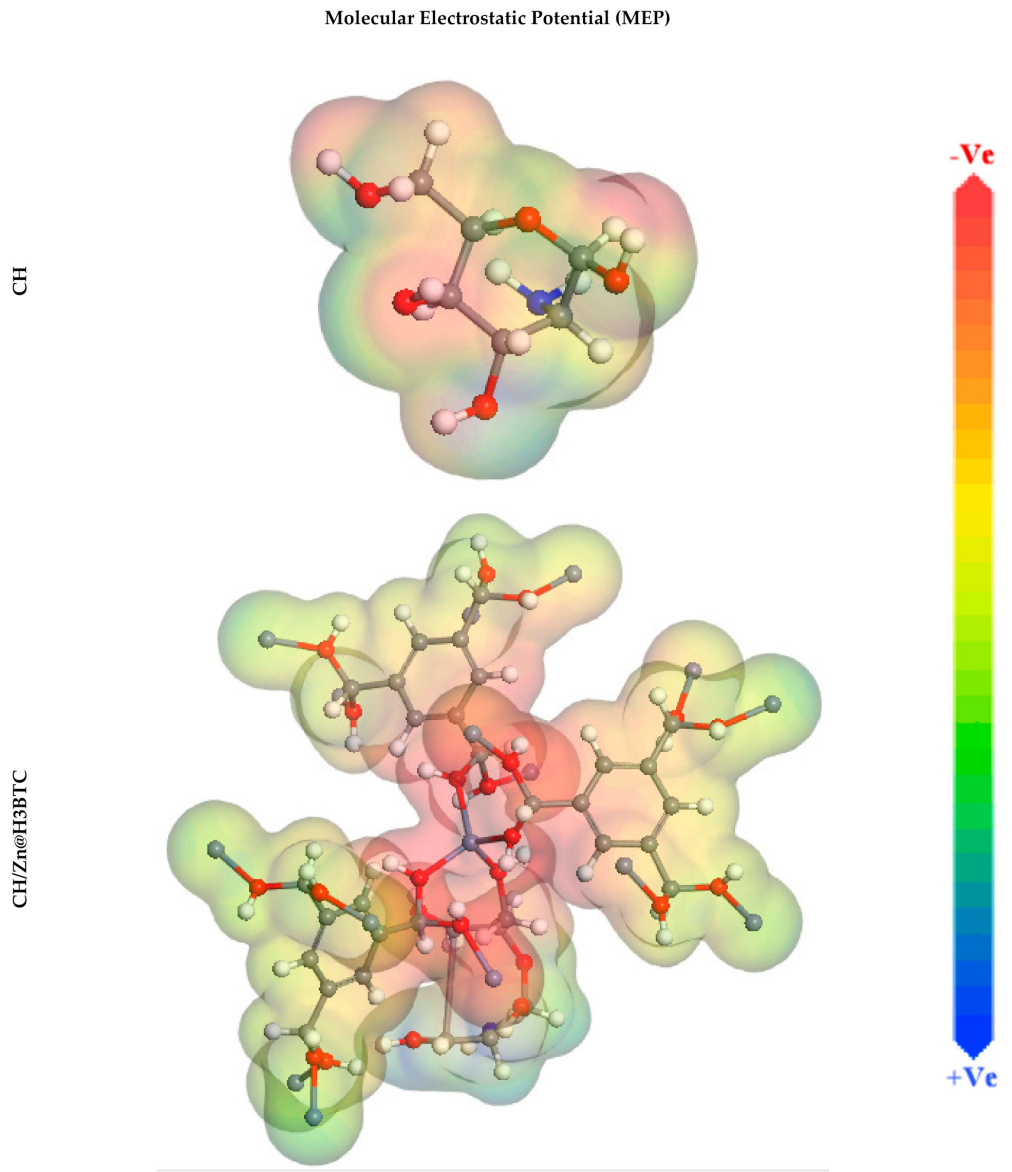
Graphical presentation of the MEP of the monomer structures of CH and CH/Zn@H_3_BTC created using the DMol3 module.

We used molecular electrostatic potential mapping (MEP) to investigate the higher-energy areas on CH/Zn@H_3_BTC and CH molecules. MEP mapping is a 3D visual descriptor used to recognize the total electrostatic effect on a molecule by the total charge distribution [66]. In the MEP maps shown in Figure 14, the red indicates the highest electron density; the MEP is always more negative (nucleophilic reaction). The blue indicates the highest positive region (electrophilic reaction) [67]. An optical examination of Figure 14 shows that the greatest negative regions were mostly above O and N atoms; however, lower electron density can be observed over the Zn atoms and phenyl moieties in the investigated molecules. These positions with high electron density (i.e., red areas) in the investigated molecules are the best sites for interactions within steel for fabricating a strongly adsorbed protective film.

### 3.6. MC Simulations

**Figure 15:**
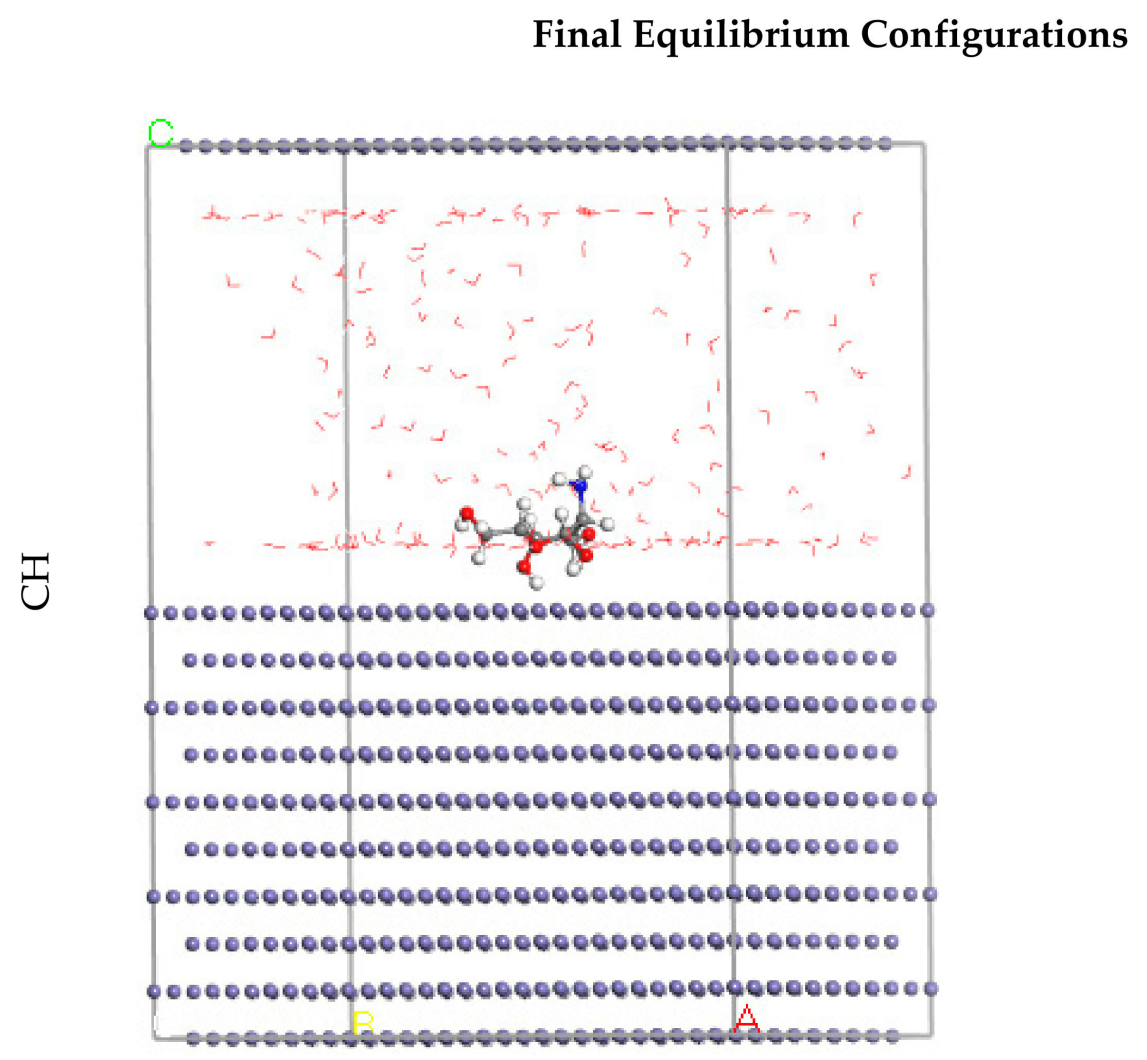

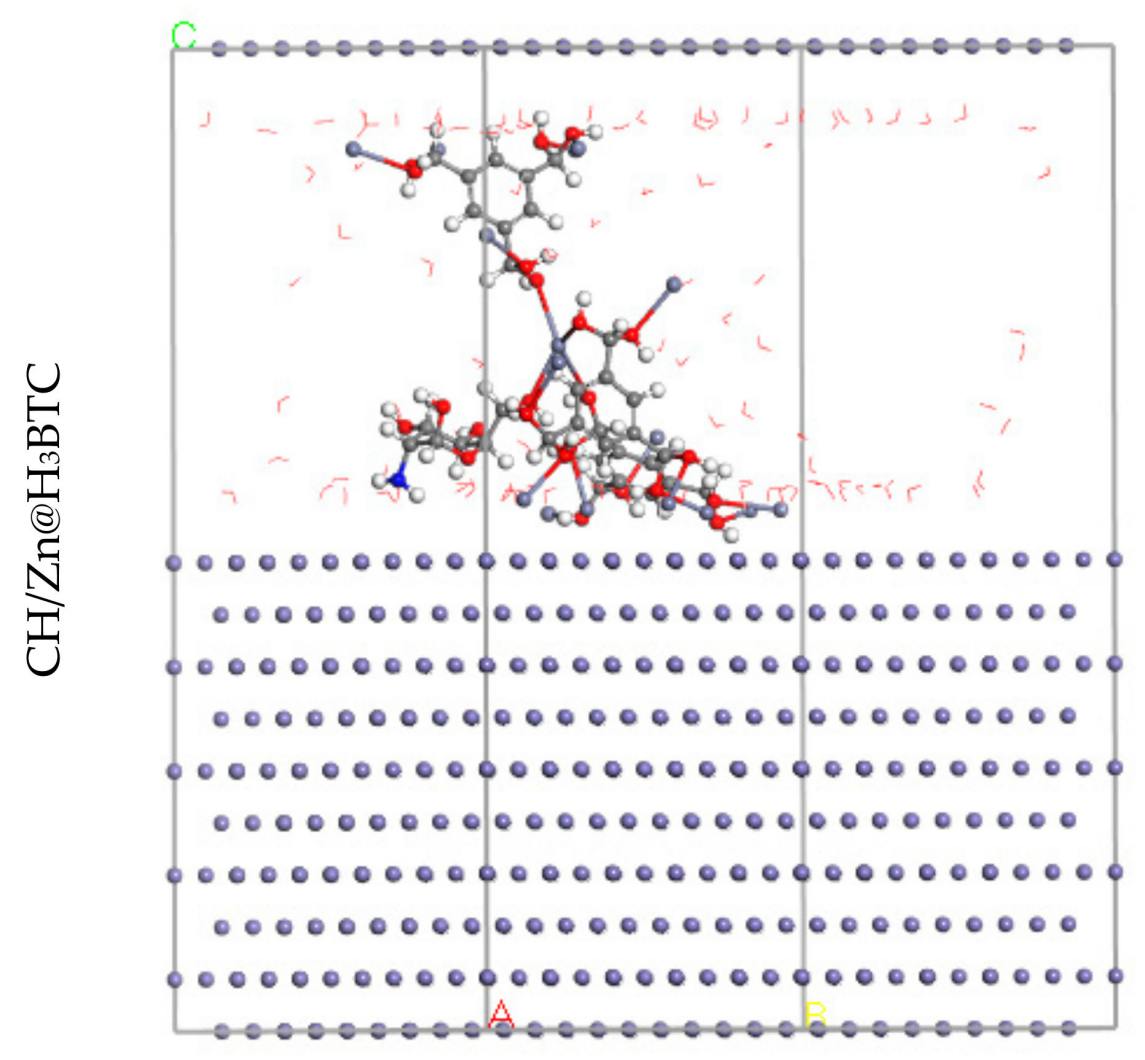
The most suitable configuration for adsorption of the monomer structures of CH and CH/Zn@H_3_BTC on Fe (1 1 0) substrate obtained by adsorption locator module.

MC simulations were used to describe the adsorption mechanism and the correlations of the examined molecules with the steel surface. Figure 15 reveals the highest appropriate adsorption formations for the CH and CH/Zn@H_3_BTC molecules on the steel surface produced with the adsorption locator module; they present an almost flat disposition, signifying an improvement in the adsorption and maximum surface coverage [68]. Moreover, the results of the adsorption energies from the MC simulations are displayed in Table 4. The data revealed that CH/Zn@H_3_BTC (−9676.99 kcal mol^−1^) has a greater negative adsorption energy value than CH (−3045.58 kcal mol^−1^), indicting the powerful adsorption of CH/Zn@H_3_BTC on the steel surface, creating a strongly adsorbed barrier that inhibits the corrosion of steel. These obtained data agree with the experimental results [69]. Additionally, Table 4 shows that the energy adsorption value of CH/Zn@H_3_BTC in the pre-geometry optimization stage. i.e., unrelaxed (−4675.19 kcal mol^−1^), was more negative than that of CH (−2908.63 kcal mol^−1^). In the post-geometry optimization stage, i.e., relaxed (−5001 kcal mol^−1^), the value was greater than that of CH (−136.94 kcal mol^−1^), indicating a stronger corrosion prohibition potency of CH/Zn@H_3_BTC compared to CH.

The d*E*_ads_/d*N*_i_ values explain the metal-adsorbates’ structure energy if adsorbed when additives or water molecules are removed [70]. The d*E*_ads_/d*N*_i_ value for CH/Zn@H_3_BTC (−1388.29 kcal mol^−1^) was found to be higher than that of CH (−103.40 kcal mol^−1^), as presented in Table 4, which shows the good adsorption of CH/Zn@H_3_BTC compare to CH. Furthermore, the water (d*E*_ads_/d*N*_i_) values are close (−14.30 kcal mol^−1^), which is small compared to the CH/Zn@H_3_BTC and CH values, revealing the strong adsorption of CH/Zn@H_3_BTC and CH molecules compared to water molecules, which supports the exchanging of water molecules with CH/Zn@H_3_BTC and CH molecules. Hence, the CH/Zn@H_3_BTC and CH molecules are strongly adsorbed on the steel surface and form a preventive barrier, which prohibits corrosion of the steel surface in an acidic medium, as established by both experimental and computational examinations.

## 4. Conclusions

In this work, a novel composite of a zinc-based 1,3,5-benezenetricarboxylic acid framework Zn@H_3_BTC coated with chitosan was synthesized via the solvothermal route. FTIR, FESEM, TGA, and BET analyses showed the successful impregnation of chitosan into Zn@H_3_BTC. The fabricated CH/Zn@H_3_BTC composite was compared to as effective inhibitors of D-steel corrosion in a sulfuric acid medium using empirical and computer simulation explorations. The results obtained from PDP and EIS are in good agreement. When CH and CH/Zn@H_3_BTC concentrations were 200 ppm, the average inhibition capacity of PDP and EIS reached a maximum of 94.1% and 97.8%, and 91.8% and 94.3%, respectively. The PDP method showed that the anticorrosive action of CH/Zn@H_3_BTC composite is dose-dependent, and its anticorrosion ability is achieved due to being a mixed type inhibitor. The *j*_cor_ value declined with the increase in the CH/Zn@H_3_BTC composite dose. The *R*_ct_ at the interface of the metal/solution gradually increased as the CH/Zn@H_3_BTC composite dose increased and the *C_dl_* decreased owing to composite adsorption. CH/Zn@H_3_BTC composite naturally adsorbed on the D steel interface, in agreement with the Langmuir isotherm model, which was considered combined physical-chemical adsorption. The FESEM pictures proved a smoother metal interface with the inclusion of 200 ppm CH and CH/Zn@H_3_BTC composite compared to the blank medium without inhibitor, due to a compact CH/Zn@H_3_BTC composite film forming on the D steel’s surface. The mechanism of the protection process was confirmed by DFT and MD modeling methods. The computational modeling techniques substantiate the obtained empirical findings. The findings showed that CH/Zn@H_3_BTC composite is an efficient inhibitor of steel corrosion in acidic corrosive solutions.

## Figures and Tables

**Table 1:** PDP parameters achieved for duplex steel in 2.0 M H_2_SO_4_ solution in the absence and presence of various concentrations of CH and CH/Zn@H_3_BTC at 50 °C.

Inhibitors Code	*C*_inh_ ppm by weight	*E*_cor_ V vs. Ag/AgCl	*j*_cor_ µA cm^−2^ ±SD	*β*_a_ mV dec ^−1^ ±SD	*−β*_c_ mV dec ^−1^ ±SD	*θ*	*ɀ*_PP_ %
Blank	0.0	−0.441	1137.7 ± 71	70.7 ± 5.2	138.8 ± 11.6	-	-
CH	20	−0.433	713.33 ± 51.4	81.5 ± 3.4	146.4 ± 10.1	0.373	37.3
	40	−0.437	550.68 ± 32.2	78.1 ± 5.7	147.9 ± 9.7	0.516	51.6
	80	−0.429	293.52 ± 19.3	79.8 ± 4.1	143.7 ± 6.8	0.742	74.2
	120	−0.442	120.59 ± 9.8	81.2 ± 7.1	140.3 ± 8.5	0.894	89.4
	200	−0.437	67.12 ± 5.3	83.9 ± 2.9	153.4 ± 6.3	0.941	94.1
CH/Zn@H_3_BTC	20	−0.442	613.22 ± 45.6	84.6 ± 3.8	152.5 ± 7.6	0.461	46.1
	40	−0.441	427.77 ± 23.9	86.1 ± 4.8	153.6 ± 12.2	0.624	62.4
	80	−0.434	145.62 ± 10.2	79.2 ± 3.9	149.2 ± 13.4	0.872	87.2
	120	−0.428	71.67 ± 6.4	87.5 ± 7.5	150.4 ± 9.8	0.937	93.7
	200	−0.427	25.02 ± 1.5	82.3 ± 7.1	153.7 ± 7.4	0.978	97.8

**Table 2:** EIS parameters achieved for duplex steel in in 2.0 M H_2_SO_4_ solution in the absence and presence of various concentrations of CH and CH/Zn@H_3_BTC at 50 °C.

Additive Codes	*C_inh._* ppm	*R*_e_ Ω cm^2^	*R*_ct_ Ω cm^2^ ±SD	*C_dl_* F cm^−2^ × 10^−6^	*R*_p_ Ω cm^2^ ±SD	*R*_L_ Ω cm^2^	*L* H cm^2^	*Q*_CPE_		*χ*^2^ *×* 10^−4^	*ζ*_E_ %
								*Y*_0_ μΩ^−1^ s*^n^* cm^−2^	*n*		
2.0 M H_2_SO_4_	0.0	0.69	20.6 ± 1.6	540.5	9.6	18.2	11.82	133.93	0.739	3.91	--
CH	20	0.83	30.4 ± 2.1	161.8	14.2	26.9	29.91	38.27	0.829	4.93	32.2
	40	0.99	41.1 ± 3.2	134.3	19.1	35.7	36.25	32.42	0.845	4.96	49.8
	80	1.21	75.4 ± 4.7	84.8	35.3	66.7	57.15	20.13	0.841	4.99	72.6
	120	1.42	129.3 ± 9.4	44.4	62.2	120.1	107.53	10.62	0.849	5.25	84.1
	200	1.78	252.4 ± 16.8	26.9	117.8	220.9	178.45	6.37	0.865	5.06	91.8
CH/Zn@H_3_BTC	20	0.83	33.3 ± 1.4	121.5	15.7	29.9	40.38	28.77	0.849	5.48	38.1
	40	0.91	61.9 ± 3.6	82.8	29.5	56.6	58.26	19.61	0.862	5.44	66.7
	80	1.26	102.8 ± 6.2	67.7	49.3	94.8	71.79	15.84	0.897	5.18	79.9
	120	1.85	177.1 ± 16.9	37.6	82.7	155.5	130.75	8.92	0.856	5.14	88.3
	200	2.24	364.8 ± 23.1	14.7	168.3	312.7	244.02	3.72	0.889	5.32	94.3

**Table 3:** The calculated quantum chemical parameters for the monomer structures of CH and CH/Zn@H_3_BTC.

Inhibitor	CH	CH/Zn@H_3_BTC
*E*_HOMO_, eV	−5.00	−9.11
*E*_LUMO_, eV	0.81	−8.57
∆*E*, eV	5.80	0.55
*I*	5.00	9.11
*A*	−0.81	8.57
*χ*	2.09	8.84
*η*	2.90	0.27
*σ*	0.34	3.66
Dipole moment value, Debye	6.09	53.16
Molecular surface area, Å^2^	193.50	1188.75

**Table 4:** Data and descriptors calculated by Mont Carlo (MC) simulation for adsorption of the monomer structures of CH and CH/Zn@H_3_BTC on Fe (1 1 0).

Structures	Adsorption Energy kcal mol^−1^	Rigid Adsorption Energy kcal mol^−1^	Deformation Energy kcal mol^−1^	d*E*_ads_/d*N*_i_: Inhibitor kcal mol^−1^	d*E*_ads_/d*N*_i_: Water kcal mol^−1^
Fe (110)	−3045.58	−2908.63	−136.94	−103.40	−14.31
CH					
water					
Fe (110)	−9676.99	−4675.19	−5001.80	−1388.29	−14.28
CH/Zn@H_3_BTC					
water					

## Data Availability

The raw/processed data generated in this work are available upon request from the corresponding author.
